# Psychosis Prognosis Predictor: A continuous and uncertainty‐aware prediction of treatment outcome in first‐episode psychosis

**DOI:** 10.1111/acps.13754

**Published:** 2024-09-18

**Authors:** Daniël P. J. van Opstal, Seyed Mostafa Kia, Lea Jakob, Metten Somers, Iris E. C. Sommer, Inge Winter‐van Rossum, René S. Kahn, Wiepke Cahn, Hugo G. Schnack

**Affiliations:** ^1^ Brain Center, Department of Psychiatry, University Medical Center Utrecht Utrecht University Utrecht the Netherlands; ^2^ Donders Institute for Brain, Cognition and Behaviour Radboud University Nijmegen the Netherlands; ^3^ Department of Cognitive Science and Artificial Intelligence Tilburg University Tilburg the Netherlands; ^4^ Early Episodes of SMI Research Center National Institute of Mental Health Klecany Czech Republic; ^5^ Department of Psychiatry and Medical Psychology, 3rd Faculty of Medicine Charles University Prague Czech Republic; ^6^ Department of Psychiatry, University Medical Center Groningen University of Groningen Groningen the Netherlands; ^7^ Department of Psychiatry Icahn School of Medicine at Mount Sinai New York City USA; ^8^ Institute of Language Sciences Utrecht University Utrecht the Netherlands

**Keywords:** psychosis prognosis prediction, machine learning, uncertainty‐aware decision making, precision psychiatry

## Abstract

**Introduction:**

Machine learning models have shown promising potential in individual‐level outcome prediction for patients with psychosis, but also have several limitations. To address some of these limitations, we present a model that predicts multiple outcomes, based on longitudinal patient data, while integrating prediction uncertainty to facilitate more reliable clinical decision‐making.

**Material and Methods:**

We devised a recurrent neural network architecture incorporating long short‐term memory (LSTM) units to facilitate outcome prediction by leveraging multimodal baseline variables and clinical data collected at multiple time points. To account for model uncertainty, we employed a novel fuzzy logic approach to integrate the level of uncertainty into individual predictions. We predicted antipsychotic treatment outcomes in 446 first‐episode psychosis patients in the OPTiMiSE study, for six different clinical scenarios. The treatment outcome measures assessed at both week 4 and week 10 encompassed symptomatic remission, clinical global remission, and functional remission.

**Results:**

Using only baseline predictors to predict different outcomes at week 4, leave‐one‐site‐out validation AUC ranged from 0.62 to 0.66; performance improved when clinical data from week 1 was added (AUC = 0.66–0.71). For outcome at week 10, using only baseline variables, the models achieved AUC = 0.56–0.64; using data from more time points (weeks 1, 4, and 6) improved the performance to AUC = 0.72–0.74. After incorporating prediction uncertainties and stratifying the model decisions based on model confidence, we could achieve accuracies above 0.8 for ~50% of patients in five out of the six clinical scenarios.

**Conclusion:**

We constructed prediction models utilizing a recurrent neural network architecture tailored to clinical scenarios derived from a time series dataset. One crucial aspect we incorporated was the consideration of uncertainty in individual predictions, which enhances the reliability of decision‐making based on the model's output. We provided evidence showcasing the significance of leveraging time series data for achieving more accurate treatment outcome prediction in the field of psychiatry.

**Significant outcomes**

- We built a model for predicting multiple outcomes that can handle time series data; the model's performance improved when receiving more information over time.
- The model incorporates the uncertainty of individual predictions in the decision‐making process; we demonstrated that this results in safer prognostic decisions.
- Models with these properties (multi‐task, time series based, considering uncertainty) can be translated into prediction tools for clinical practice.

**Limitations**

- Due to the design of the OPTiMiSE trial, the sample size for predictions at week 10 was up to four times smaller compared to for predictions at week 4.
- Further validation on external datasets is needed.
- All patients in the dataset used amisulpride; generalization of our model to patients receiving other treatments needs to be investigated.

## 1 INTRODUCTION

There is an abundance of research into predictors of outcome in psychosis, but until now, clinicians are unable to reliably predict the disease course nor the success rate of (pharmacological) treatment intervention(s) of an individual patient. A possible way forward is the use of machine learning techniques.^1^, ^2^ In psychiatry research, machine learning techniques are increasingly being used, particularly in psychotic disorders.^3^ Several studies^4^, ^5^, ^6^, ^7^, ^8^, ^9^, ^10^, ^11^, ^12^, ^13^, ^14^ examined illness progress in existing psychotic disorders, each predicting different outcomes. Of these, two studies^4^, ^14^ aimed to predict antipsychotic treatment response. In an open‐label randomized clinical trial of five broadly used antipsychotics (*N* = 334),^4^ clinical and sociodemographic variables were used as inputs to a support vector machine to predict the level of functioning at four and 52 weeks after the start of antipsychotic treatment in patients with first‐episode psychosis with an accuracy of 71%–72%. Another study^14^ predicted response to asenapine in a double‐blind, placebo‐controlled trial including 532 patients, and found that early improvement of several individual symptoms predicted treatment response with an accuracy of 78%–85%.

The aforementioned psychosis prognosis prediction studies have noteworthy limitations that hinder their practical use as prediction tools in day‐to‐day clinical practice. Firstly, the importance of different outcomes may vary for individual patients, and therefore, clinicians and patients should have the ability to choose the relevant outcomes to be predicted. However, most existing prediction models in psychosis research are single‐task models, focusing on predicting only one outcome measure. To overcome this limitation, we employ a multi‐task learning^15^ approach in our study, training a model to predict multiple outcome measures simultaneously.

Secondly, in clinical practice, it is crucial to have an adaptive prediction tool that can accommodate the changes in a patient's status and incorporate additional information obtained during each visit. Traditional machine learning methods used in psychosis prognosis prediction lack the ability to accommodate the dynamic nature of patients' status. To address this limitation, we propose employing a machine learning approach capable of making predictions based on multiple assessments over time. One such approach is long short‐term memory (LSTM), a type of recurrent neural network that has been successfully used in various healthcare domains.^16^

Thirdly, as machine learning models can be uncertain about their prediction (like human beings), the clinician needs to be informed about the uncertainty involved in model predictions. Knowing that the model is (very) sure about a certain prediction, the clinician may more confidently integrate the machine's prediction with their own judgment. On the other hand, when the model is unsure about a certain prediction, the clinician may opt not to use the machine's prediction as a guide to treat the patient. To date, most models used for treatment outcome prediction do not incorporate the uncertainty in estimated model parameters (i.e., the epistemic uncertainty^17^) into the model predictions, so it is unclear how far we can trust their predictions. Therefore it is desirable to integrate the model uncertainty in the predictions to facilitate the clinical usage of the model and to allow for more trustworthy decision‐making.^18^, ^19^ Such an improvement eventually results in safer prediction models and will reduce the risk of making wrong decisions, for example, by tapering or switching antipsychotic medication too early or unnecessarily late.

In our study, we present a machine learning framework that predicts multiple outcomes based on longitudinal patient data while integrating prediction uncertainty to facilitate more reliable clinical decision‐making. This prediction model was trained using data from the OPTiMiSE study, an international multicenter prospective clinical research trial.^20^ By addressing aforementioned limitations (which are discussed in detail in the Supporting Information  S12), we aim to enhance the applicability and trustworthiness of prediction models in guiding clinical practice and optimizing treatment strategies for patients with psychosis.

## 2 MATERIALS AND METHODS

### 2.1 The Psychosis Prognosis Predictor

Treatment of a first‐episode patient is a sequence of (re)evaluation of the patient's status and effects of treatment thus far and decisions about (changing) treatment. At each time point, the psychiatrist integrates newly available data with information gathered in the past. For it to be useful in this clinical practice, a machine‐learning prediction tool must do the same. In this study, the functioning of the prediction model is evaluated in the OPTiMiSE study.^20^

### 2.2 The dataset

The OPTiMiSE study^20^ is a large, international, multicenter antipsychotic three‐phase switching study. The study was conducted in 27 sites in 14 European countries and Israel. Patients with first‐episode psychosis were examined at multiple visits and treated with antipsychotic medication that could be changed based on the patient's response. We used data from patients in phase one and phase two. In the first phase, patients (*N* = 446/371 started/completed) were treated with amisulpride (up to 800 mg/day) for four weeks. Patients who then met the criteria for symptomatic remission did not continue to the next phase. Patients not in remission went on to phase two (*N* = 93/72 started/completed) and either continued using amisulpride or switched to olanzapine (≤20 mg/day) for six weeks. Patient characteristics at the start of each treatment phase are shown in Table 1.

**TABLE 1:** Patient characteristics at the start of each treatment phase.

	Phase one (*N* = 446)	Phase two (*N* = 93)
Age (years)	26.0 (6.0)	25.2 (5.4)
Sex		
Women	134 (30%)	23 (25%)
Men	312 (70%)	70 (75%)
Race		
White	386 (87%)	86 (92%)
Other	60 (13%)	7 (8%)
Education (years)^a^	12.3 (3.0)	11.9 (2.7)
Living status		
Independently	83 (19%)	20 (22%)
With assistance	363 (81%)	73 (78%)
Employment status		
Employed or student	185 (41%)	33 (35%)
Unemployed	261 (59%)	60 (65%)
Disease type^b^		
Schizophreniform disorder	190 (43%)	28 (30%)
Schizoaffective disorder	27 (6%)	2 (2%)
Schizophrenia	229 (51%)	63 (68%)
Comorbid major depressive disorder	34/429 (8%)	9/91 (10%)
Suicidality	55/429 (13%)	10/91 (11%)
Substance abuse or dependence in the past 12 months	75/429 (17%)	9/91 (10%)
Type of care at baseline		
Inpatient	276 (62%)	53 (57%)
Outpatient	170 (38%)	40 (43%)
Duration of untreated psychosis (months)	6.3 (6.2)	8.4 (7.3)
Antipsychotic naïve	187 (42%)	54 (58%)
Clinical scores^c^		
PANSS total score	78.2 (18.7)	85.7 (16.4)
PANSS Positive subscale	20.2 (5.5)	21.7 (5.1)
PANSS Negative subscale	19.4 (7.1)	22.4 (7.0)
PANSS General subscale	38.6 (9.8)	41.6 (9.3)
CGI severity^d^	4.5 (0.9)	4.7 (0.8)
Depression score^e^	13.5 (4.6)	14.2 (4.8)
BMI	23.4 (5.0)	23.9 (4.3)

*Note*: Values are mean (sd), *n* (%), or *n*/*N* (%) (because of incomplete data).

Abbreviations: BMI, body‐mass index (kg/m^2^); CGI, clinical global impression; PANSS, Positive and Negative Syndrome Scale.

^a^ In school from age 6 years onwards.

^b^ According to the Mini International Neuropsychiatric Interview (suicidality: medium to high suicide risk).

^c^ Scores range from 30 to 210 (total score), 7–49 (positive and negative scale), and 16–112 (general scale); high scores indicate severe psychopathology.

^d^ Scores range from 1 to 7; high scores indicate increased severity of illness.

^e^ According to the Calgary Depression Scale for Schizophrenia. Scores range from 0 to 27; high scores indicate increased depression.

### 2.3 Outcome measures and predictors

Our primary outcome measure for prediction was symptomatic remission. Secondary outcome measures were clinical global remission and functional remission. Symptomatic remission was defined the same way as in the OPTiMiSE study, according to the consensus criteria of Andreasen et al.^21^ based on the Positive And Negative Syndrome Scale (PANSS),^22^ albeit without the minimum duration of six months. For global illness, we used the Clinical Global Impression (CGI) scale.^23^ We considered a CGI score of 4 or lower as clinical global remission. For the functional outcome, we used the Personal and Social Performance (PSP) scale. We considered a global PSP score of 71 points or higher as functional remission, following Morosini's definition where a global PSP score from 71 to 100 points refers only to mild difficulties.^24^ For an overview of all features from the OPTiMiSE study that are used as predictors in our model, see Table 2.

**TABLE 2:** The type, number, and list of features from the OPTiMiSE study that are used as predictors in our model.

Module	Type	Number of features	Features
Static input features	Demographic	20	Age (con), Sex (bin), Race (cat), Immigration status (bin), Marital status (bin), Divorce status (bin), Occupation status (bin), Occupation type (cat), Previous occupation status (bin), Previous occupation type (cat), Father's occupation (cat), Mother's occupation (cat), Years of education (con), Highest education level (cat), Father's highest degree (cat), Mother's highest degree (cat), Living status (bin), Dwelling (cat), Income source (cat), Living environment (cat)
	Diagnostic	7	DSM‐IV classification (cat), Duration of the current psychotic episode (con), Current psychiatric treatment (cat), Psychosocial interventions status (bin), Estimated prognosis (cat), Hospitalization status (bin)
	Lifestyle	7	Recreational drugs history (bin), Recreational drugs since last visit (bin), Caffeine drinks per day (con), Last caffeine drink (cat), Drink Alcohol (bin), Alcoholic drinks in the last year (cat), Smoking status (bin)
	Somatic	11	Height (con), Weight (con), Waist (con), Hip (con), BMI (con), Systolic blood pressure (con), Diastolic blood pressure (con), Pulse (con), ECG abnormality (bin), Last mealtime (cat), Last meal type (cat)
	Treatment	1	Average medication dosage (con)
	CDSS	9	Calgary Depression Scale for Schizophrenia (con)
	SWN	20	Subjective well‐being under Neuroleptic Treatment Scale (con)
	MINI	67	Mini International Neuropsychiatric Interview (bin)
Dynamic input features	PANSS	30	Positive And Negative Symdrome Scale (con)
	PSP	5	Personal and Social Performance Scale (con)
	CGI‐S	2	Clinical Global Impression Scale severity and improvement (con)

Abbreviations: bin, binary measure; cat, categorical measure; con, continuous measure.

Patients were assessed at various time points during different phases of the study. These assessments included baseline (week 0, W_0_), the end of phase one (week four, W_4_), and the end of phase two (week ten, W_10_), as well as additional assessments at weeks one, two, six, and eight (W_1_, W_2_, W_6_, and W_8_, respectively). These frequent assessments allow for a comprehensive evaluation of a patient's status and enable the tracking of changes over time. By incorporating data from these multiple time points, our study aims to capture the dynamic nature of the disease and improve the accuracy of psychosis prognosis prediction.

### 2.4 The design of the Psychosis Prognosis Predictor

**FIGURE 1:**
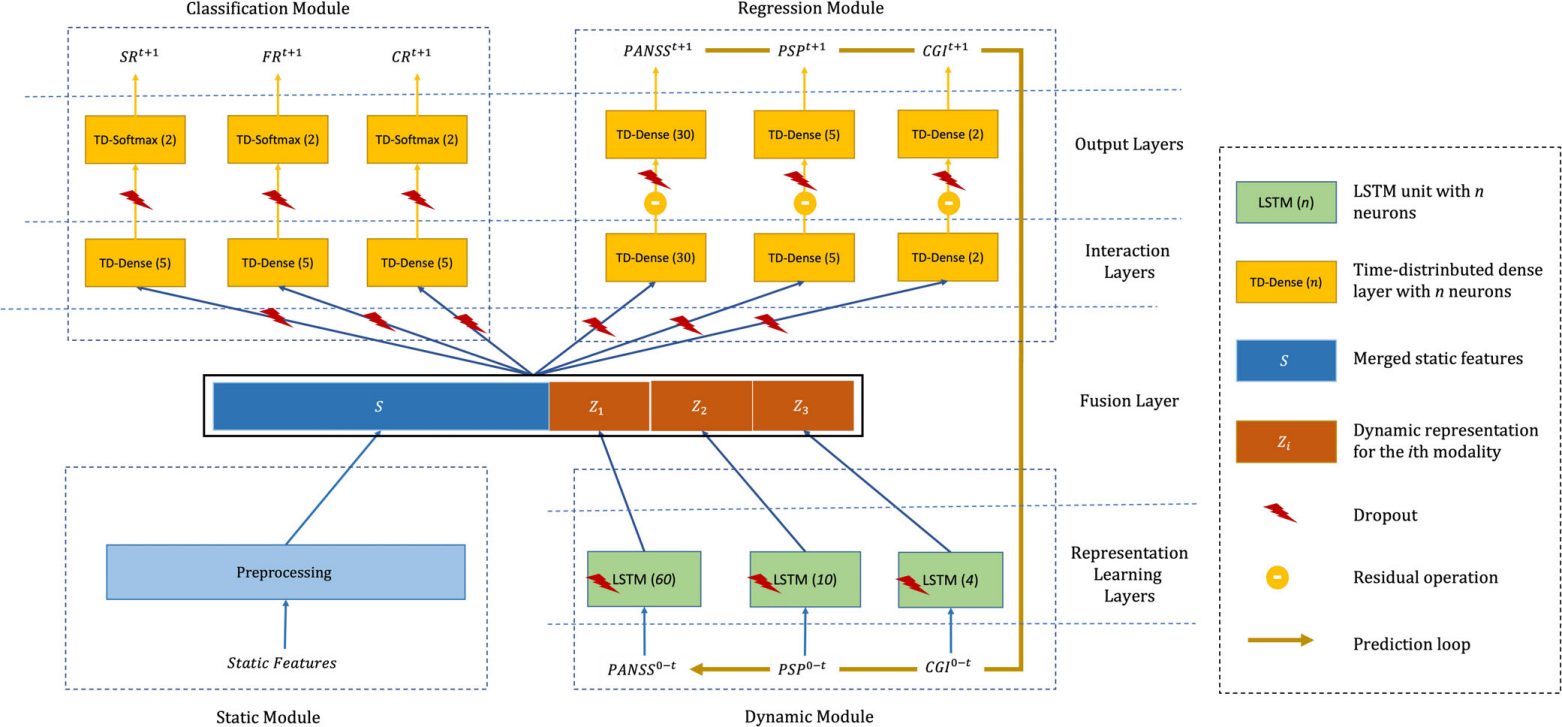
The psychosis prognosis predictor architecture consists of four layers that are organized into four conceptual modules. The layers include (1) the representation learning layer that learns a middle representation for dynamic features; (2) the fusion layer that merges the preprocessed static features with dynamic middle representations; (3) the interaction layer that seeks to benefit from interaction between static features and dynamic features from different modalities; (4) the output layer that predicts the outputs at the next time step. The modules include (1) the static module for preprocessing and merging the static features; (2) the dynamic module that includes LSTM units for learning middle representation for dynamic features from time *0* to time *t*; (3) the regression module for predicting the dynamic measures at the next time step (*t + 1*), and (4) classification module for predicting the outcomes (CR, clinical global remission; FR, functional remission; SR, symptomatic remission) at the next time step. The prediction loop from the output of the regression module to the inputs of the dynamic module (the thick yellow arrow) enables the network to predict the outcomes at an arbitrary future point.

We introduce a multi‐modal, time‐aware, and multi‐task recurrent neural network architecture designed specifically for psychosis prognosis prediction. This architecture is capable of handling multi‐modal data from various sources, capturing the dynamic nature of the data as it evolves over time, and simultaneously predicting multiple outcome measures. The proposed architecture, depicted in Figure 1, comprises four conceptual modules that work synergistically to predict the outcomes (for a detailed description about the model architecture, see Supporting Information S12):

1. *Static module*: which receives input features that are not changing over time (i.e., the static features, see Table 2) and preprocesses them by imputing the missing values, scaling the continuous features, and one‐hot encoding of categorical features.
2. *Dynamic module*: which receives input features that change over time (i.e., the dynamic features, see Table 2). This module includes modality‐specific LSTM units, a recurrent neural network architecture,^25^ that is well suited for making predictions on time series data.^26^, ^27^ Each LSTM unit transfers the dynamic features from baseline (W_0_) to a user‐defined endpoint *t*, into a time‐varying middle representation.
3. *Regression module*: which receives the outputs of the static and dynamic modules to predict the dynamic data at the next time point *t* + 1. The predicted outputs can be concatenated to the dynamic inputs at time point *t* and earlier, and fed again to the dynamic module for predicting the measures at *t + 2*. This recursive procedure can be employed for predicting the outcomes in the unlimited future.
4. *Classification module*: which receives the same inputs as the regression module and predicts the probability of target classes (not‐remitted or remitted) at time *t +* 1 for three outcome measures (symptomatic remission, clinical global remission, and functional remission).

### 2.5 From predictions to uncertainty‐aware clinical decisions

In general, the probabilities predicted by a classifier are used as outcomes for clinical decision‐making, by discretizing the probabilities into classes of decisions by imposing a hard threshold (e.g., 0.5 in binary classification). However, a classifier, like a human being, can sometimes be unsure about its predictions. How sure a model is about its predictions can be quantified by incorporating the epistemic uncertainty,^17^, ^28^ that is, the uncertainty in the model parameters, into its predictions. Now, the challenge is to combine the predicted probabilities and their estimated uncertainties into final clinical decisions.

In this paper, we use a fuzzy logic^29^ approach for translating the predictions of the model into uncertainty‐aware clinical decisions. Fuzzy logic provides a mathematical framework for representing vague and imprecise information. We employ Mamdani's rule‐based fuzzy inference procedure.^30^ Using five fuzzy membership functions, the predicted class probabilities are combined with their associated uncertainties, and then are transformed to one out of seven clinical decisions, namely ‘definite no‐remission (DN)’, ‘probable no‐remission (PN)’, ‘unsure no‐remission (UN)’, ‘unsure (US)’, ‘unsure remission (UR)’, ‘probable remission (PR)’, and ‘definite remission (DR)’; (see Supporting Information S12 for a detailed description of the procedure).

**FIGURE 2:**
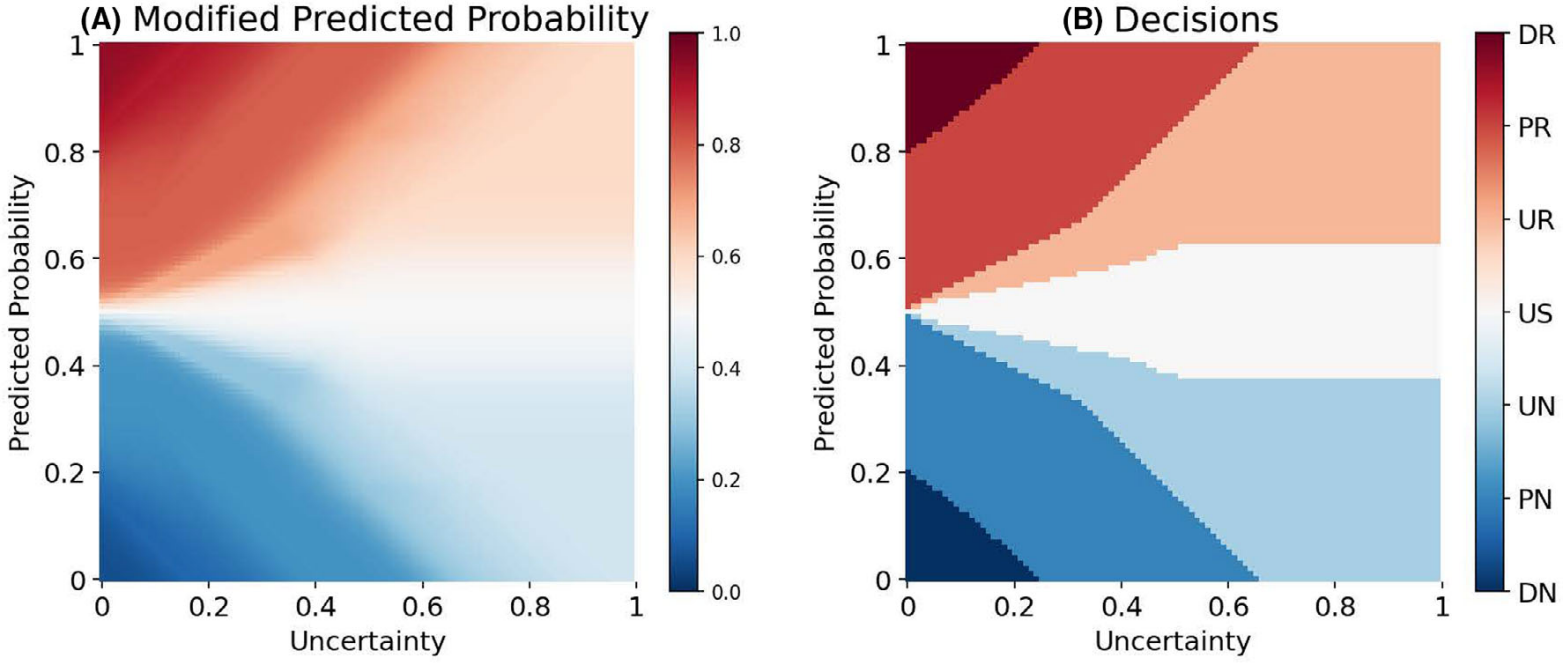
(A) The modified probability of symptomatic remission (color scale) after adjusting the predicted probability (y‐axis) based on model uncertainty (x‐axis). For example, *p* = 1.00 (i.e., the model predicts 100% remission for a certain patient) can be transferred to a value between 0.60 and 1.00 depending on the level of model uncertainty. This modification resulted in better‐calibrated predictions of the probability of remission and is thus more suitable for clinical usage. Calibration of the prediction models is a critical factor but often neglected,^31^, ^32^ especially in identifying the threshold of risks in clinical decision‐making.^33^ (B) The span of decision surfaces for seven categories of clinical decisions. The fuzzy logic approach for decision‐making enables the prediction model to also say “I do not know” when the decision lies in the unsure (US) category. This is a crucial feature for more safe applications of ML models in clinical settings.^18^ Furthermore, psychiatrists can refrain from relying on model predictions when the decisions lie in the unsure remission (UR) or unsure no‐remission (UN) to reduce the risk of wrong decisions. The other categories are ‘definite no‐remission (DN)’, ‘probable no‐remission (PN)’, ‘probable remission (PR)’, and ‘definite remission (DR)’.

Figure 2A shows how the fuzzy logic framework modifies the predicted probability of remission based on the estimated model uncertainty. Figure 2B shows how the decision surface is divided between seven categories of decisions. These uncertainty‐aware categorical decisions can play the role of meta‐information aiding clinicians in more safer AI‐aided decision‐making. For example, if a decision lies in one of the “unsure” categories, the clinicians can ignore the model prediction and rely on other sources of information (e.g., a second opinion from a colleague or gathering more information about the patient).

### 2.6 Model training procedure and evaluation

For more robust training of a complex model on small data, we pretrained the model on synthetic data and used data augmentation techniques (see Supporting Information S12). Furthermore, we used dropout in the proposed neural network architecture, with a two‐fold advantage: during the training, it prevents the network from overfitting^34^; while in the prediction phase, it enables estimating the uncertainty in the predictions.^35^ The estimated uncertainties are used in the proposed decision‐making module (see section *From predictions to uncertainty‐aware clinical decisions*) to translate the model predictions of outcomes into risk‐aware clinical decisions.

In this study, the classification performance of the proposed architecture was evaluated using 20 repetitions of two cross‐validation strategies: (1) 10‐fold cross‐validation and (2) one‐site‐out cross‐validation. The repeated cross‐validation procedures helped account for variations with data perturbation and ensure reliable estimates of the model's generalization performance. For each repetition of the cross‐validation, evaluation metrics were calculated to measure the classification performance. The metrics used in this study include:

1. *Area Under the Receiver Operating Characteristic Curve* (*AUC*): quantifies the overall discriminative power of the model. It represents the ability of the model to distinguish between the positive and negative classes.
2. *Balanced Accuracy* (*BAC*)
3. *Sensitivity*
4. *Specificity*

### 2.7 Experimental setup

**FIGURE 3:**
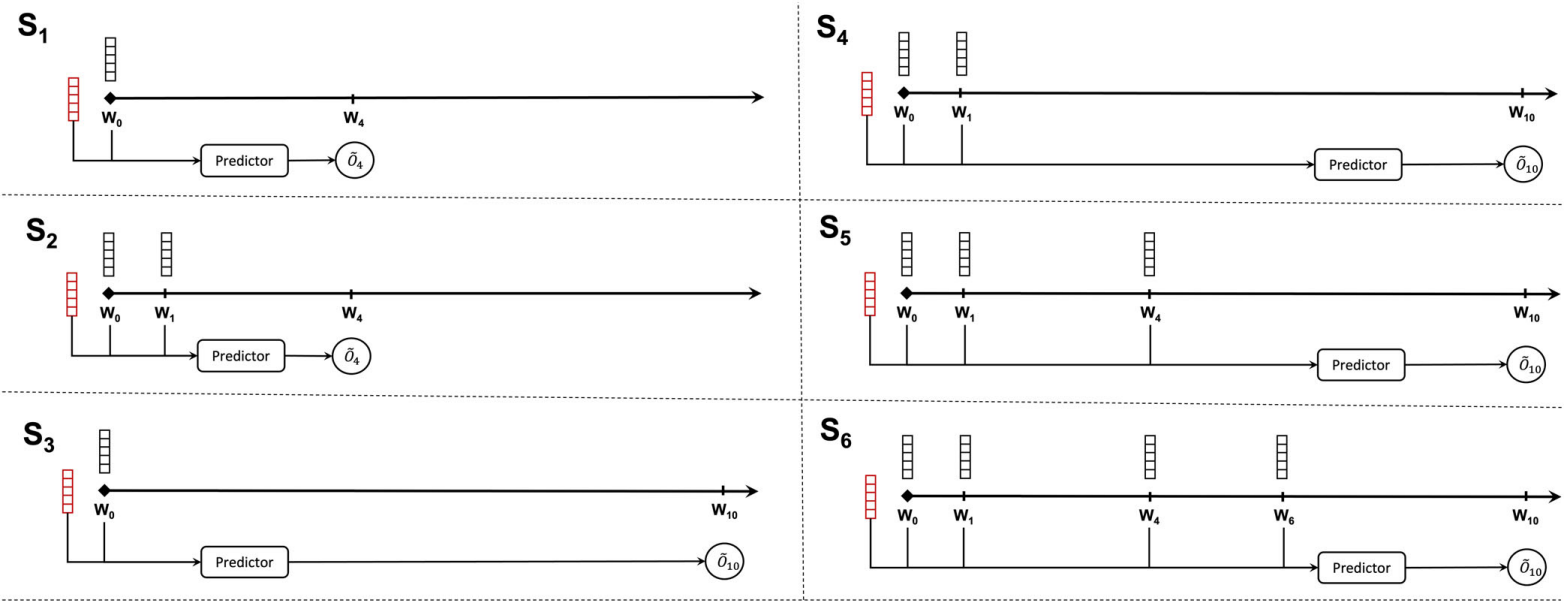
Prognosis prediction models in six clinical scenarios. Static and dynamic features are represented as red and black blocks, respectively. (S_1_) Only information from W_0_ is used for predicting the outcome at W_4_. (S_2_) Prediction at W_4_ is made after a 1‐week follow‐up by adding the dynamic information from W_1_. (S_3_) Only information from W_0_ is used for predicting the outcome at W_10_. (S_4_) Prediction at W_10_ is made after a 1‐week follow‐up by adding the dynamic information from W_1_. (S_5_) All the information from the first phase of the study is used to predict the outcome at W_10_. (S_6_) The dynamic information from W_6_ is also used as input data to the model for prediction at W_10_.

We use the model to predict the outcomes at four weeks (W_4_) and ten weeks (W_10_) following the initiation of treatment (W_0_). In order to assess the impact of including patient status information obtained during the treatment phase on the accuracy of the predictions, we conducted a performance comparison of the predictor when using different lengths of data points over time, ranging from W_1_ to W_6_ (as illustrated in Figure 3). This evaluation was carried out across six distinct clinical scenarios (S_1–6_): Predicting W_4_‐outcomes based on data at W_0_ (S_1_) or W_0_ + W_1_ (S_2_), and predicting W_10_‐outcomes based on data at W_0_ (S_3_), W_0_ + W_1_ (S_4_), W_0_ + W_1_ + W_4_ (S_5_), or W_0_ + W_1_ + W_4_ + W_6_ (S_6_) (see Figure 3), allowing us to examine the predictive capabilities of the model under various conditions.

## 3 RESULTS

### 3.1 More data over time results in higher prediction accuracy

**FIGURE 4:**
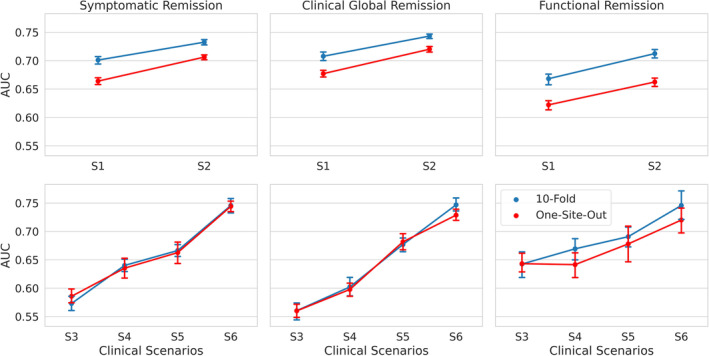
AUCs of the model across three outcome measures (first column: Symptomatic remission, second column: Clinical global remission, and third column: Functional remission) for six clinical scenarios. The x‐axes represent the clinical scenarios in phase one (S_1_ and S_2_) and phase two of the study (S_3_, S_4_, S_5_, and S_6_). The y‐axis shows the AUC. The blue and red lines represent the results for 10‐fold and one‐site‐out cross‐validation, respectively. The error bars show the standard deviation of performance across 20 repetitions. The results in the first row show the AUCs in phase one in a 4‐week prediction. The added use of time point W_1_ increases the AUC for all outcome measures, with added AUC ranging from 0.03 to 0.05. The second row shows the results of phase two in a 10‐week prediction. Except for one instance (input: W_0_–W_1_, outcome: Functional remission; validation: One‐site out), each added time point further increases the prediction performance for all outcome measures.

As summarized in Table 3 and Figure 4, using one‐site‐out cross‐validation, AUC for the 4‐week outcomes in S_1_ and S_2_ scenarios ranged from 0.66 for functional remission to 0.71 for symptomatic remission. For the 10‐week outcomes, AUC ranged from 0.72 for functional remission to 0.74 for symptomatic remission (for balanced accuracy, sensitivity and specificity, see sTables 1‐3 in the Supplementary Tables S11 and sFigures [Link], [Link], [Link]). Across all outcome measures, the AUC of the 4‐week predictions improved by 0.04–0.05 when not only baseline data (W_0_) but also data after one week (W_1_) were used. For 10‐week predictions, the use of all time series data improved AUC by 0.08–0.17, across all outcome measures.

**TABLE 3:** Performance of the prediction models predicting three outcome measures (symptomatic remission, clinical global remission, and functional remission) for six clinical scenarios (S_1_–S_6_).

Clinical Scenario	*N*	Symptomatic remission		Clinical global remission		Functional remission	
AUC		10‐fold	One‐site‐out	10‐fold	One‐site‐out	10‐fold	One‐site‐out
S_1_	371	0.701 (0.015)	0.664 (0.014)	0.708 (0.018)	0.677 (0.014)	0.668 (0.021)	0.622 (0.019)
S_2_	371	0.733 (0.011)	0.706 (0.010)	0.743 (0.009)	0.720 (0.011)	0.712 (0.018)	0.662 (0.018)
S_3_	72	0.573 (0.031)	0.586 (0.029)	0.560 (0.035)	0.560 (0.028)	0.642 (0.056)	0.643 (0.039)
S_4_	72	0.640 (0.025)	0.635 (0.043)	0.602 (0.038)	0.598 (0.027)	0.669 (0.045)	0.641 (0.052)
S_5_	72	0.666 (0.025)	0.663 (0.043)	0.677 (0.028)	0.682 (0.032)	0.691 (0.042)	0.678 (0.074)
S_6_	72	0.746 (0.030)	0.744 (0.022)	0.747 (0.028)	0.729 (0.024)	0.746 (0.059)	0.720 (0.053)

*Note*: Performance is measured by the area under the receiver operating characteristic curve (AUC). The values are averaged over 20 repetitions of 10‐fold and one‐site‐out cross‐validation. The values in the parentheses represent the standard deviation over these repetitions. S_1_ and S_2_: although 446 subjects entered phase one of the study, due to dropout of 75 subjects during this phase, the number of subjects used in these models is 371. S_3_–S_6_: 250 subjects achieved symptomatic remission after phase one (and therefore did not continue to phase two), and there was an additional dropout of 28 subjects between phase one and two. Although thus 93 subjects entered phase two, due to dropout of 21 subjects during this phase, the number of subjects used in these models is 72.

### 3.2 Incorporating model uncertainty reduces the risk of decision making

To quantitatively evaluate the advantage of using uncertainty‐aware predictions, we evaluated the prediction accuracy for symptomatic remission in six clinical scenarios at four levels of conservativeness:

- Level 0, in which the trivial threshold‐based approach is used for decision‐making. A hard threshold of 0.5 is applied to the predicted probability of remission to decide between non remission (below the threshold) or remission (equal or above the threshold) decisions. In fact, the proposed decision‐making method is not used.
- Level 1, in which the clinician abstains from utilizing the model's predictions that lie in the ‘unsure (US)’ category (when the model says “I do not know”).
- Level 2 of conservativeness, where predictions from the three most uncertain prediction categories (US, UR, and UN) are not used for clinical decision‐making.
- Level 3, where in the most conservative usage of model predictions, only the most certain decisions of the model (the DR and DN categories) are employed by the clinicians for decision‐making.

**FIGURE 5:**
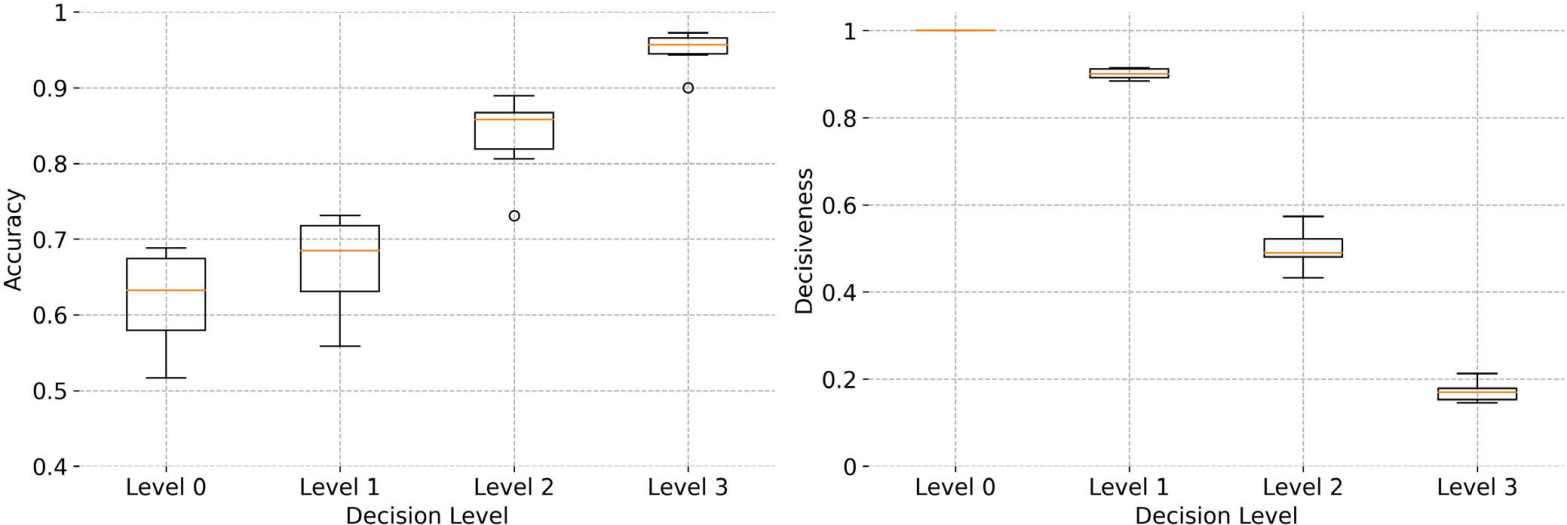
A comparison between the accuracy and decisiveness of ML‐aided decision‐making at four levels of conservativeness (0–3). The boxplots represent the average accuracy (left) and decisiveness (right) over 20 repetitions of 10‐fold cross‐validation across six clinical scenarios. Using more certain predictions for decision‐making results in less decisive but more accurate models.

The results of applying increasing levels of conservativeness are presented in Table 4. An incremental trend in the accuracy of decisions is seen, when rising the conservativeness level from 0 to 3 (see also Figure 5), which is, naturally, accompanied by a decrease in the number of patients for whom an ML‐aided decision is made (represented by decisiveness in the table). At level 1, and by excluding ~10% of decisions in the US category, the accuracy of the model is improved by ~0.06 across all clinical scenarios. At level 2, excluding ~50% (the uncertain predictions in US, UR, and UN) from decision‐making, results in a further increase in accuracy to ~0.86. By restricting the decision‐making to DR and DN categories at level 3, the accuracy of the model is increased to ~0.95 within ~16% of patients with decisions. Thus, the clinicians can trust the DR and DN decisions with 0.95 confidence (although without being able to use decisions for ~84% of their patients). This is a crucial feature for more trustworthy decision‐making in clinics because the users (i.e., clinicians) not only receive an ML‐aided data‐driven recommendation from the machine but are also informed about the risk involved in relying on these predictions. The more confidence in the model's predictions (in DR and DN categories), the less risk is involved in AI aided decision‐making.

**TABLE 4:** The decisiveness (the proportion of decided sample to total sample) and the accuracy of decision for symptomatic remission, for six clinical scenarios (rows) and four decision levels (columns).

Clinical scenario	Decisiveness				Accuracy			
	Level 0	Level 1	Level 2	Level 3	Level 0	Level 1	Level 2	Level 3
S_1_	1.00 (0.00)	0.88 (0.02)	0.48 (0.03)	0.15 (0.02)	0.65 (0.02)	0.70 (0.02)	0.86 (0.02)	0.97 (0.01)
S_2_	1.00 (0.00)	0.90 (0.02)	0.53 (0.03)	0.18 (0.02)	0.69 (0.01)	0.73 (0.01)	0.87 (0.01)	0.97 (0.01)
S_3_	1.00 (0.00)	0.91 (0.03)	0.57 (0.08)	0.21 (0.06)	0.52 (0.03)	0.56 (0.02)	0.73 (0.05)	0.90 (0.04)
S_4_	1.00 (0.00)	0.91 (0.02)	0.50 (0.04)	0.16 (0.04)	0.57 (0.03)	0.62 (0.02)	0.81 (0.02)	0.94 (0.02)
S_5_	1.00 (0.00)	0.89 (0.04)	0.43 (0.06)	0.15 (0.05)	0.61 (0.03)	0.67 (0.04)	0.86 (0.03)	0.95 (0.02)
S_6_	1.00 (0.00)	0.91 (0.03)	0.48 (0.05)	0.18 (0.04)	0.68 (0.03)	0.72 (0.03)	0.89 (0.03)	0.96 (0.02)
Median	1.00	0.90	0.48	0.16	0.61	0.67	0.86	0.95

*Note*: The values are averaged over 20 repetitions of 10‐fold cross‐validation. The values in the parentheses represent the standard deviation over these repetitions.

## 4 DISCUSSION

This study set out to build a prediction model that has the potential to be used as a tool to assist in clinical decision‐making. To the best of our knowledge, this is the first model that fulfills three crucial criteria for use in clinical practice (for a more detailed comparison between our approach and more common machine learning models, see Supporting Information S12). Firstly, by using a recurrent neural network architecture, the model was trained on time series data. Previous studies^4^, ^5^, ^6^, ^7^, ^8^ only used baseline data in their prediction models, thus, do not accommodate the use of time series data in the same prediction model. In contrast, the proposed architecture provides the flexibility to add additional data over time as the status of the patient develops after receiving a certain treatment. Therefore, unlike other prediction models, it better fits real‐world data,^36^ where a patient is a dynamic entity and assessed regularly.

Secondly, our architecture is multi‐task, so one prediction model can predict multiple outcome measures simultaneously. This feature has been highlighted as important by our patient and doctor panel of advisors which regularly contributes to the understanding of real‐world patient and doctor needs. The involvement of such panels was found to be crucial in building trust in AI solutions in healthcare, among both patients and doctors.^37^ In previous studies, when predicting multiple outcome measures, separate prediction models were needed, one for each outcome measure^4^, ^5^, ^6^, ^7^, ^38^, ^39^, ^40^, ^41^, ^42^, ^43^, ^44^, ^45^; In this study, we were able to predict symptomatic, clinical global and functional remission in just one model. The multi‐task model provides a way for clinicians and their patients to know what the predicted (differential) effects of treatment are in several domains.

Thirdly, we used the uncertainty of predictions to adjust the prediction accuracy and this was implemented in a novel decision‐making module. Thus, clinicians and their patients will get additional information about how sure the machine is about an individual prediction. As a result, this improves the chance of making the right treatment decision. We consider this an important feature, given the potential consequences of wrong decision‐making in treatment. For example in psychosis, unnecessary side effects of antipsychotic medication or longer duration of untreated psychosis are to be considered in this aspect. Models that have predictive uncertainty incorporated will help to create more trust with the physicians (and patients) using them. Furthermore, the ability to say “I don't know” when the model is uncertain about an individual prediction, is a necesarry feature for safe translation of machine learning models to clinical practice.^18^ Using this flexible multi‐task recurrent architecture that incorporates the uncertainty of individual predictions, we took a leap forward toward improving patient care with the help of machine learning prediction models.

Considering the specific characteristics of our current solution, we found accuracies of up to 0.72 AUC for 4‐week prediction and up to 0.74 AUC for 10‐week prediction using one‐site‐out as a validation method. These results are comparable to previously conducted studies.^4^, ^5^, ^7^, ^9^ We have shown that the use of multiple time points increased the accuracy of prediction for all outcome measures for both 4‐week and 10‐week predictions.

Although the accuracy of our models is not above the 80% threshold suggested by the APA^46^ when all patients are incorporated, we still consider our models could be clinically relevant after incorporating the prediction uncertainty. When uncertain predictions are discarded (our ‘decision‐making level 2’), across the six different prediction models, predictions were still possible for 43%–57% of the patients with accuracies ranging from 0.73 to 0.89, with even five of our six models achieving an accuracy above 0.8. This feature could therefore be an important step toward reaching our goal of building an interactive tool for individual prediction of the prognosis in psychosis.

In this study, we merely used clinical and sociodemographic predictor variables, only requiring basic medical examination and questionnaires to obtain. Other studies suggest more advanced medical tests like blood serum biomarkers^47^ or structural MRI scans^48^ prove meaningful in predicting antipsychotic treatment response. Combining these different types of predictor variables in one prediction model might be an essential next step in order to attain higher prediction accuracies, which we will explore in future research. However, the feasibility of such a model in clinical practice, with a higher burden on patients due to the more invasive methods required, and the higher medical costs associated with this, is an important factor to be considered. Expanding our model with other kinds of data, specifically possibly important “easy to obtain” clinical predictors not currently available (e.g., family history, somatic comorbidity, traumatic experiences), might therefore be a more desirable way to improve accuracy and validity.

### 4.1 Limitations

Our LSTM model can use time series data, but currently, this is only possible when data from all previous time points are also available. In clinical practice, this could be a potential problem, in situations where a patient misses an appointment or is not capable of providing information in certain exams or questionnaires at some point. The risk of having missing values is bigger for models that rely on many features. Feature selection could lower this risk, but could not be reliably implemented (see the detailed Discussion in Supporting Information S12). The problem can be solved by using LSTM models that can handle missing measurements^49^ or by incorporating the length of time intervals in the modeling process.^50^ We consider these as possible future directions to extend our work.

Considering the data the model was tested on, all patients in the dataset used amisulpride in the first phase, and amisulpride or olanzapine in the second phase of their treatment. Therefore our model only applies to patients using amisulpride. Also, not all potentially relevant predictor variables were available in our data set, such as childhood adverse events. A larger dataset with a more diverse and heterogeneous sample would improve the clinical applicability of future models.

## 5 CONCLUSION

We developed and tested a psychosis prognosis prediction model that has properties that are required for use in daily clinical practice. Using a flexible multi‐task recurrent neural network architecture that was optimized for this goal, the ability to use time series data was shown to be of great importance once prediction models will be used in clinical care. By building a multi‐task model, different clinically relevant outcomes can be predicted simultaneously. For more reliable decision‐making, we built a decision‐making module that considers the uncertainty of individual predictions and we demonstrated its usefulness.

## FUNDING INFORMATION

This work was supported by ZonMw (project ID 63631 0011) and by a research grant from the AI for Health working group of the TU/e‐WUR‐UU‐UMCU (EWUU) alliance.

## CONFLICT OF INTEREST STATEMENT

RSK reports consulting fees from Alkermes, Sunovion, Gedeon‐Richter, and Otsuka.

### PEER REVIEW

The peer review history for this article is available at https://www.webofscience.com/api/gateway/wos/peer‐review/10.1111/acps.13754.

## ETHICS STATEMENT

All relevant ethical guidelines have been followed, and any necessary IRB and/or ethics committee approvals have been obtained.

## PATIENT CONSENT STATEMENT

All necessary patient/participant consent has been obtained and the appropriate institutional forms have been archived, and any patient/participant/sample identifiers included were not known to anyone (e.g., hospital staff, patients or participants themselves) outside the research group so cannot be used to identify individuals.

## CLINICAL TRIAL REGISTRATION

All clinical trials and any other prospective interventional studies must be registered with an ICMJE‐approved registry, such as ClinicalTrials.gov. Any such study reported in the manuscript has been registered.

OPTiMiSE dataset: https://www.thelancet.com/journals/lanpsy/article/PIIS2215‐0366(18)30252‐9/fulltext.

## Supporting information


**Figure S1.** The data augmentation process. A set of ten samples with time‐length 2–5 are generated for a sample with the length of five timepoints.

**Figure S2.** (a) Five Gaussian membership functions for the probability of remission. These functions are used to map the values of the probability of remission (*p*), the worst‐case probability of remission (*p*_*w*_), and the best‐case probability of remission (*p*_*b*_) in the x‐axis to a membership value (between 0 and 1) in the y‐axis for ‘very low’, ‘low’, ‘medium’, ‘high’, and ‘very high’ categories; (b) Gaussian membership functions for seven clinical decisions, ‘definite no‐remission (DN)’, probable no‐remission (PN)’, ‘unsure no‐remission (UN)’, ‘unsure (US)’, ‘unsure remission (UR)’, ‘probable remission (PR)’, ‘definite remission (DR)’.

**Figure S3.** Seven rules in the proposed fuzzy inference system for translating the predicted probability of remission (*p*), the worst‐case probability of remission (*p*_*w*_), and the best‐case probability of remission (*p*_*b*_) into risk‐aware clinical decisions. The green stars show the value of the corresponding membership function in each rule for an example prediction with *p* = 0.9, *p*_*w*_ = 0.25, and *p*_*b*_ = 1.00. The orange and blue boxes represent the fuzzy max and min operations, respectively. The gray area in the last right column shows the mass under the membership function of each decision. These masses are combined using fuzzy max aggregation. The x‐coordinate of the centroid of the aggregated mass represents the uncertainty‐aware probability of remission ($\tilde{p}$) that aggregates the model uncertainty into the final prediction.

**Figure S4.** Balanced accuracies (BACs) of the model across three outcome measures (first column: symptomatic remission, second column: clinical global remission, and third column: functional remission) for six clinical scenarios. The x‐axes represent the clinical scenarios in phase one (S_1_ and S_2_) and phase 2 of the study (S_3_, S_4_, S_5_, and S_6_). The y‐axis shows the BAC. The blue and red lines represent the results for 10‐fold and one‐site‐out cross‐validation, respectively. The error bars show the standard deviation of performance across 20 repetitions. The results in the first row show the BAC in phase one in a 4‐week prediction. The added use of time point W_1_ increases the BAC for all outcome measures. This is mainly due to the increased sensitivity of the model when a new time point is added. The second row shows the results of phase two in a 10‐week prediction. Except for one instance (functional remission), each added time point further increases the BAC for all outcome measures. The increase in the BACs in this case is a byproduct of the increased specificity (see sFigures [Link], [Link]) of the model when a new time point is added.

**Figure S5.** Sensitivity (SEN) of the model across three outcome measures (first column: symptomatic remission, second column: clinical global remission, and third column: functional remission) for six clinical scenarios. The x‐axes represent the clinical scenarios in phase one (S_1_ and S_2_) and phase two of the study (S_3_, S_4_, S_5_, and S_6_). The y‐axis shows the SEN. The blue and red lines represent the results for 10‐fold and one‐site‐out cross‐validation, respectively. The error bars show the standard deviation of SENs across 20 repetitions. The results in the first row show the SEN in phase one in a 4‐week prediction. The added use of time point W_1_ increases the SEN for all outcome measures. The second row shows the results of phase two in a 10‐week prediction. In most cases adding a new time point results in a reduced sensitivity of the model.

**Figure S6.** Specificity (SPC) of the model across three outcome measures (first column: symptomatic remission, second column: clinical global remission, and third column: functional remission) for six clinical scenarios. The x‐axes represent the clinical scenarios in phase one (S_1_ and S_2_) and phase two of the study (S_3_, S_4_, S_5_, and S_6_). The y‐axis shows the SPC. The blue and red lines represent the results for 10‐fold and one‐site‐out cross‐validation, respectively. The error bars show the standard deviation of SPCs across 20 repetitions. The results in the first row show the SPC in phase one in a 4‐week prediction. The added use of time point W_1_ slightly decreases the SPC for all outcome measures. The second row shows the results of phase two in a 10‐week prediction. In all cases adding a new time point results in higher model specificity.

**Figure S7.** To handle dynamic patient status in outcome prediction using conventional ML approaches, we need specialized models for data collected at each visit.

**Figure S8.** When using conventional ML approaches for outcome prediction, due to their fixed input size, we cannot feed them with accumulated data over time. We need a new model for the mixed data.

**Figure S9.** Using conventional approaches, we should train several specialized models to accurately predict at different time points in the future.

**Figure S10.** Using conventional single‐task approaches, we need to train one model per outcome. This is while the proposed multi‐task approach can predict several outcomes simultaneously.

**Tables S11.** Supplementary Tables.

**Text S12.** Supporting Information.

## Data Availability

All data produced in the present study are available upon reasonable request to the authors.
