# Supplementary figures and images for: Psychosis Prognosis Predictor: A continuous and uncertainty‐aware prediction of treatment outcome in first‐episode psychosis
Source: Acta Psychiatr Scand. 2024 Sep 18;151(3):280–92. doi: 10.1111/acps.13754 (PMC11787921; doi:10.1111/acps.13754)

# 1 sample before before augmentation

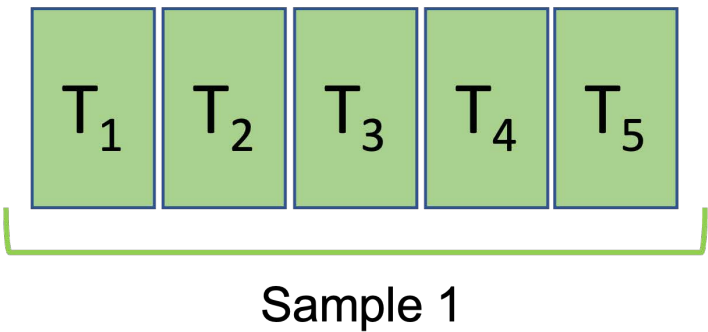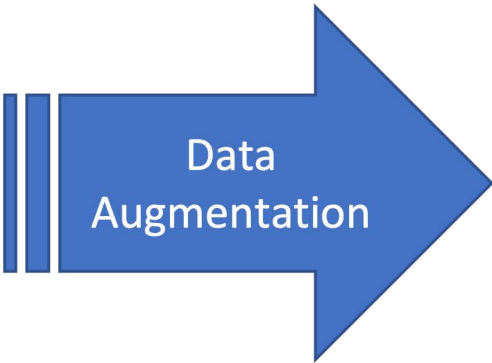

# 10 samples after augmentation

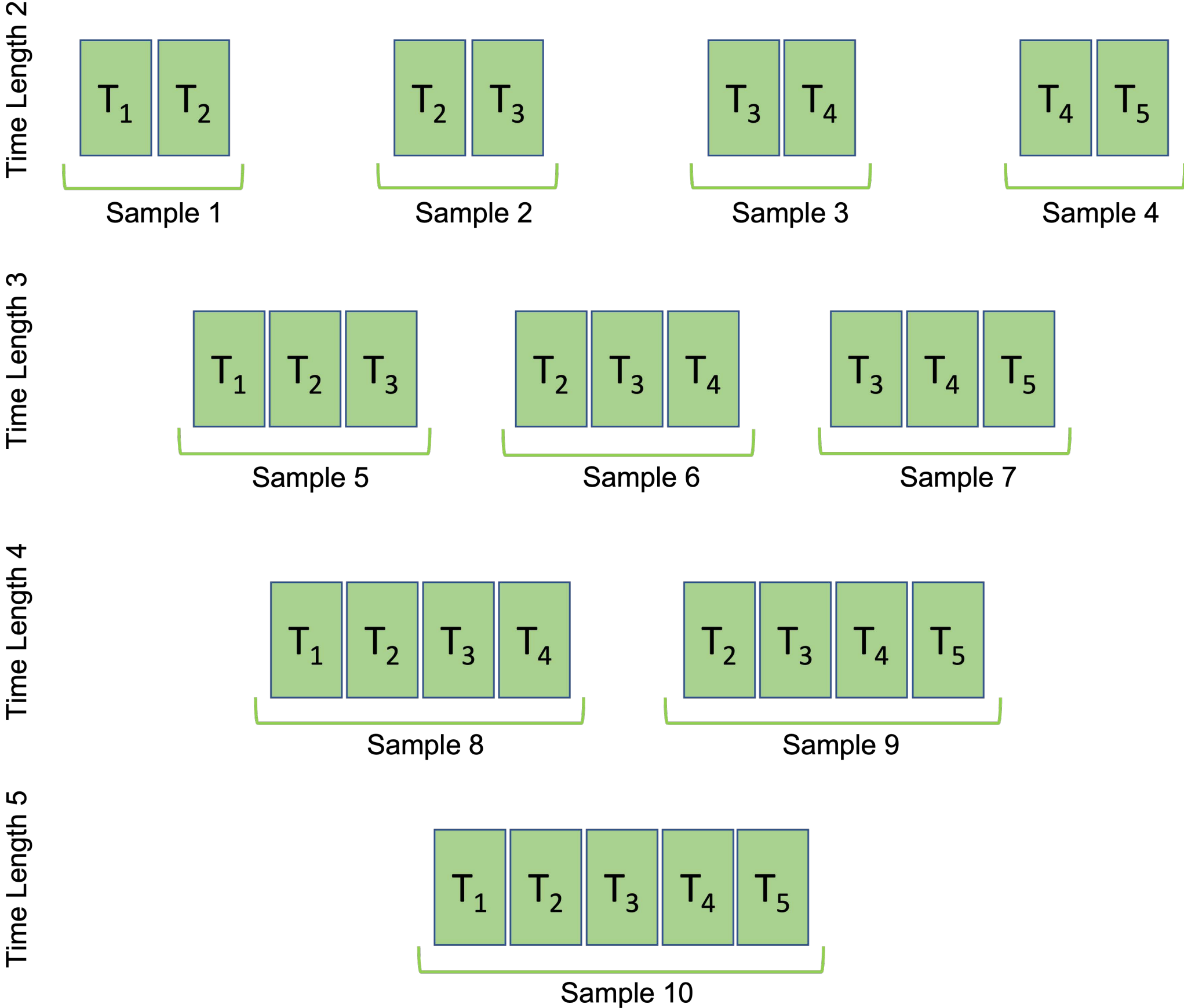

Supplement: Supplementary file 1 — Figure S1. The data augmentation process. A set of ten samples with time‐length 2–5 are generated for a sample with the length of five timepoints. [file ACPS-151-280-s003.pdf]

(a)

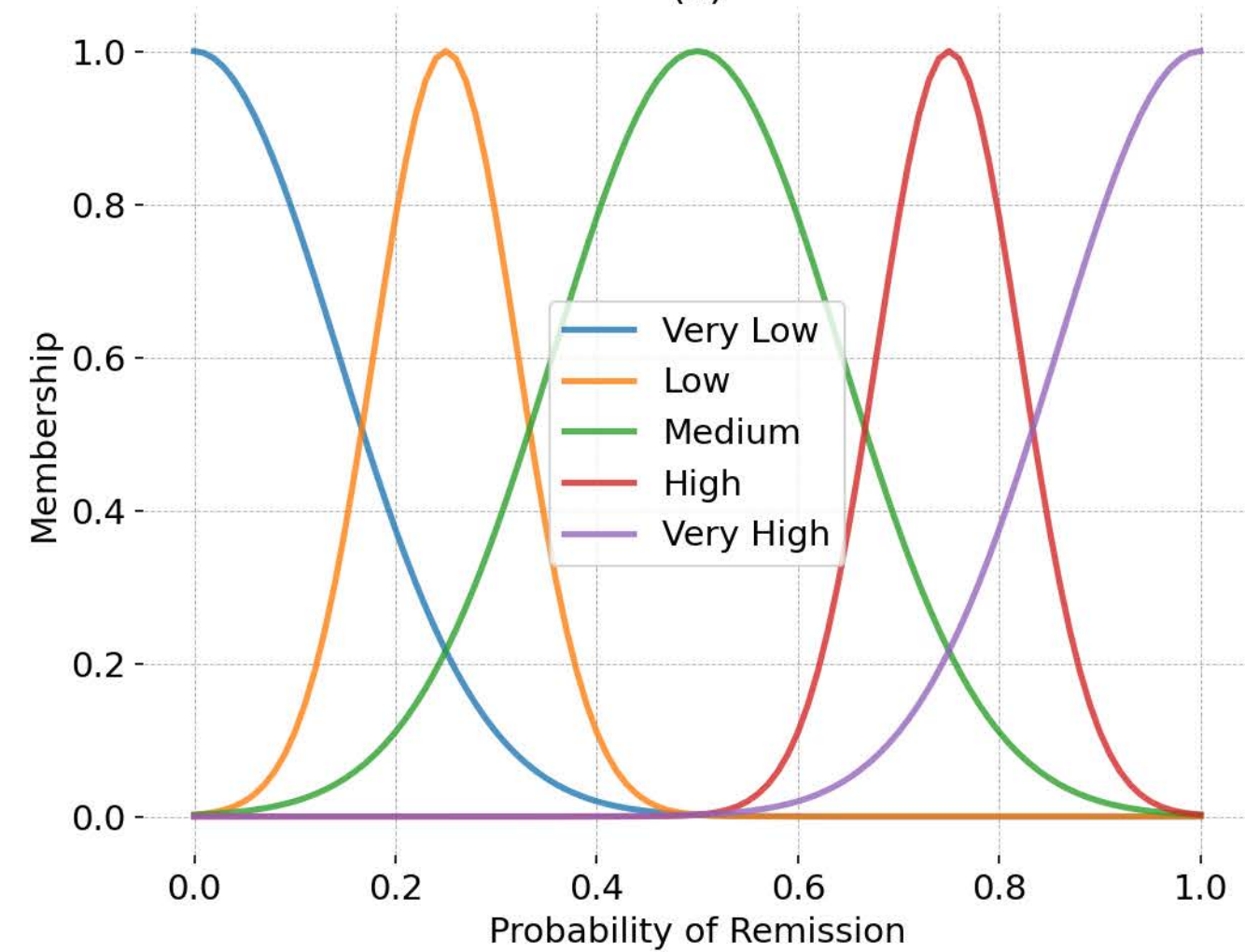

(b)

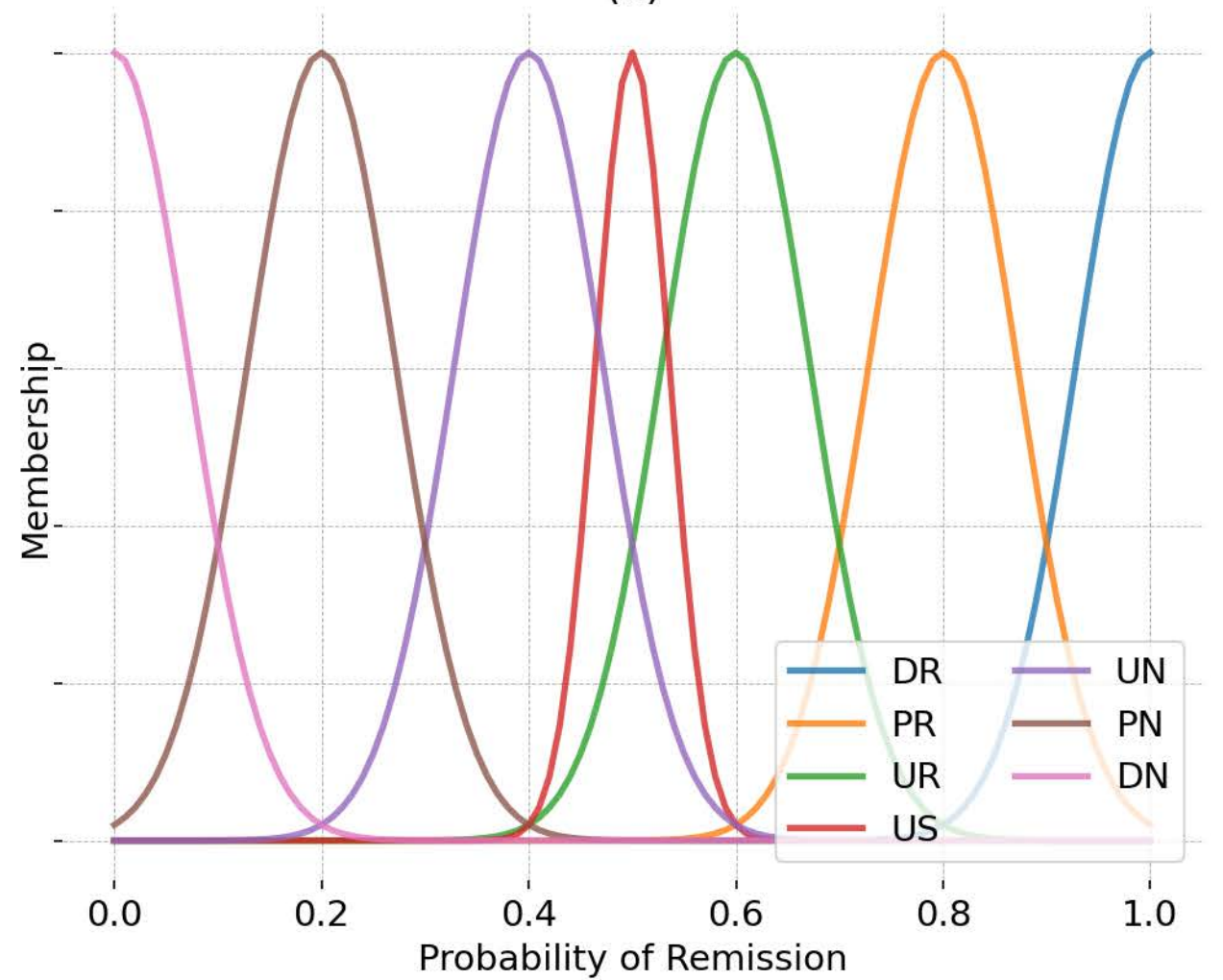

Supplement: Supplementary file 2 — Figure S2. (a) Five Gaussian membership functions for the probability of remission. These functions are used to map the values of the probability of remission (p), the worst‐case probability of remission (p w ), and the best‐case probability of remission (p b ) in the x‐axis to a membership value (between 0 and 1) in the y‐axis for ‘very low’, ‘low’, ‘medium’, ‘high’, and ‘very high’ categories; (b) Gaussian membership functions for seven clinical decisions, ‘definite no‐remission (DN)’, probable no‐remission (PN)’, ‘unsure no‐remission (UN)’, ‘unsure (US)’, ‘unsure remission (UR)’, ‘probable remission (PR)’, ‘definite remission (DR)’. [file ACPS-151-280-s001.pdf]

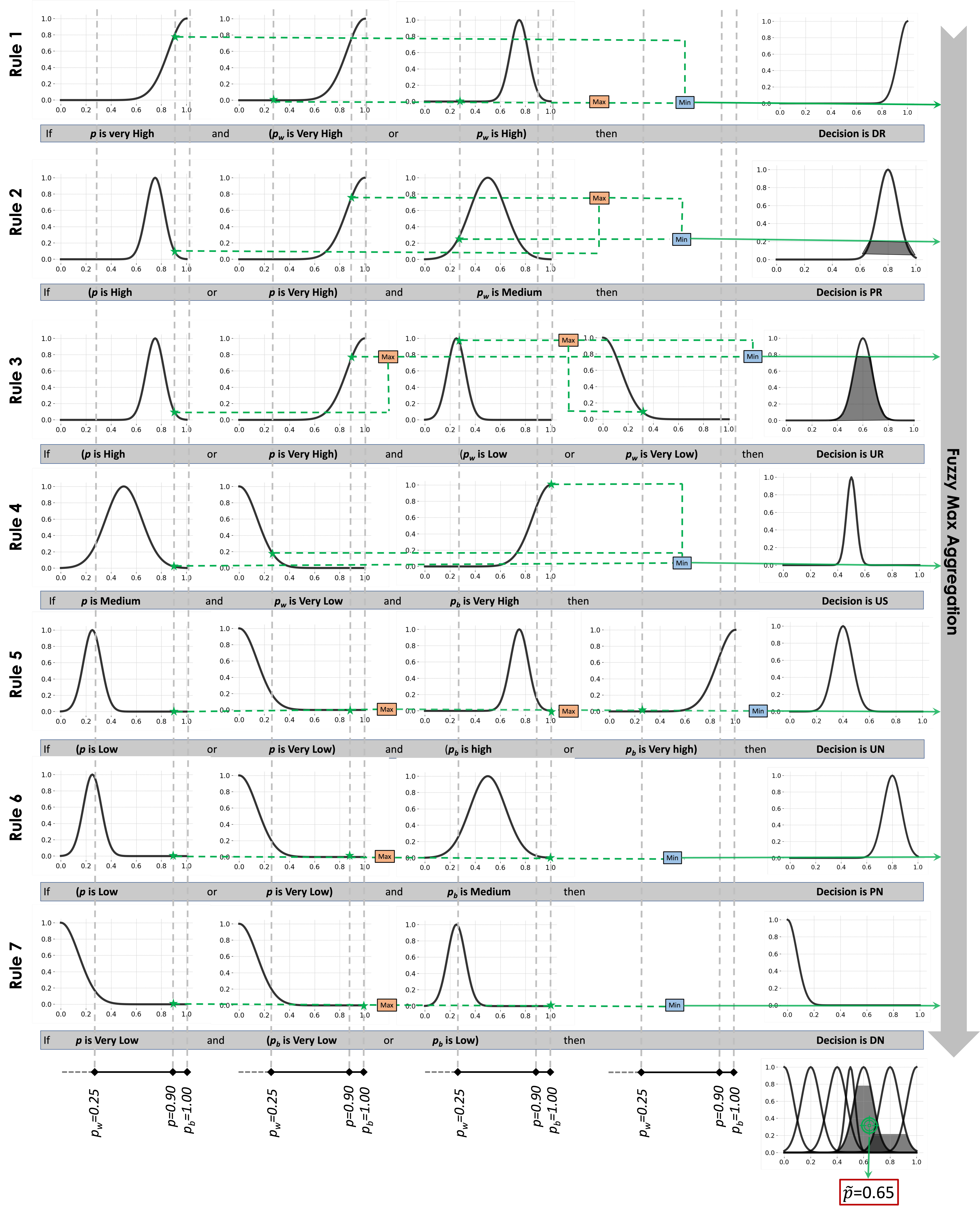

Supplement: Supplementary file 3 — Figure S3. Seven rules in the proposed fuzzy inference system for translating the predicted probability of remission (p), the worst‐case probability of remission (p w ), and the best‐case probability of remission (p b ) into risk‐aware clinical decisions. The green stars show the value of the corresponding membership function in each rule for an example prediction with p = 0.9, p w = 0.25, and p b = 1.00. The orange and blue boxes represent the fuzzy max and min operations, respectively. The gray area in the last right column shows the mass under the membership function of each decision. These masses are combined using fuzzy max aggregation. The x‐coordinate of the centroid of the aggregated mass represents the uncertainty‐aware probability of remission (p~) that aggregates the model uncertainty into the final prediction. [file ACPS-151-280-s009.pdf]

Symptomatic Remission

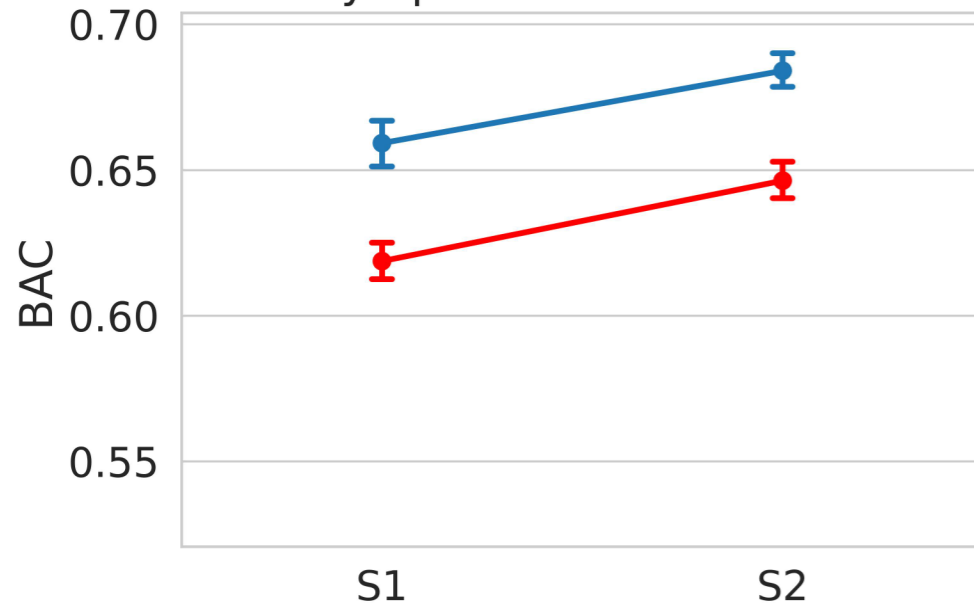

Clinical Global Remission

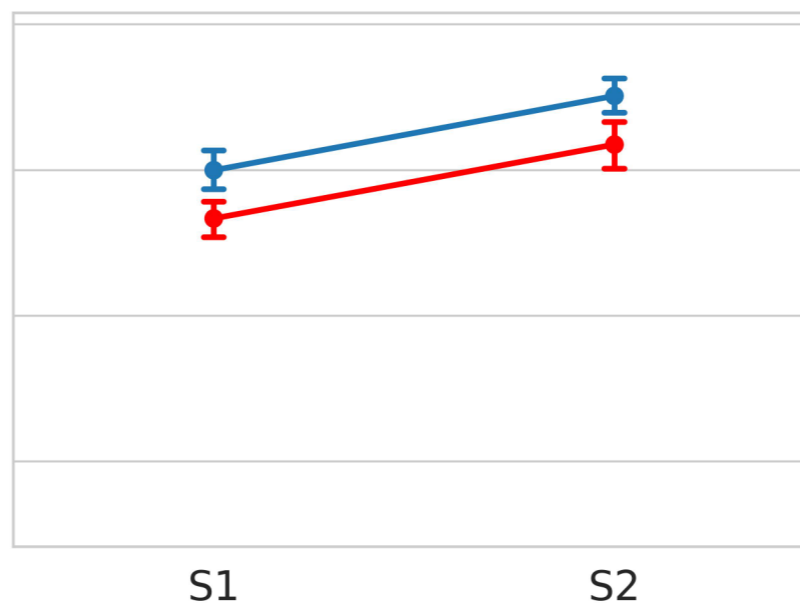

Functional Remission

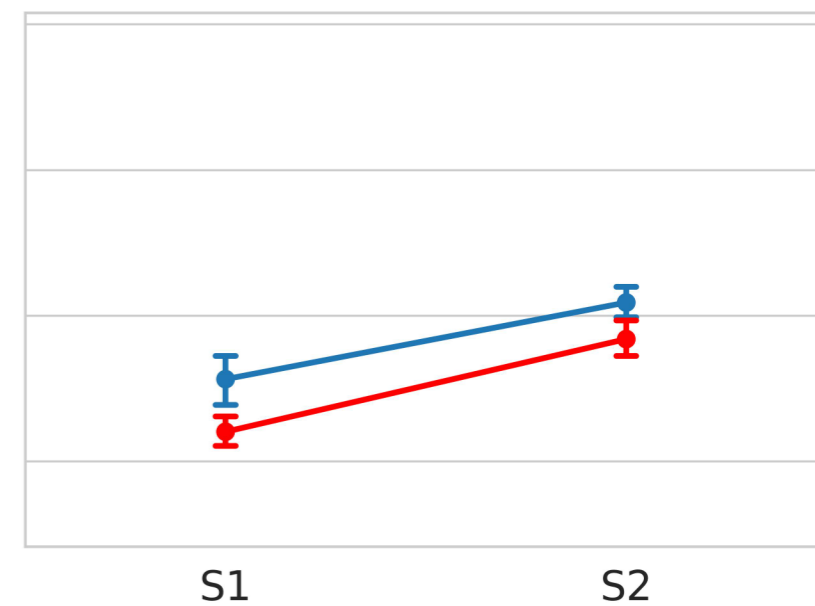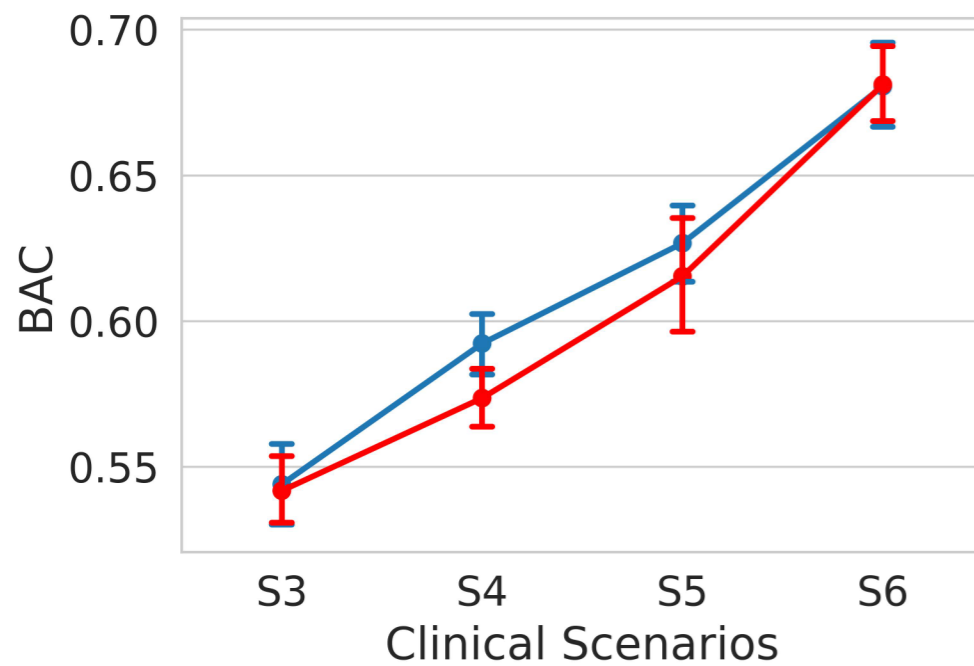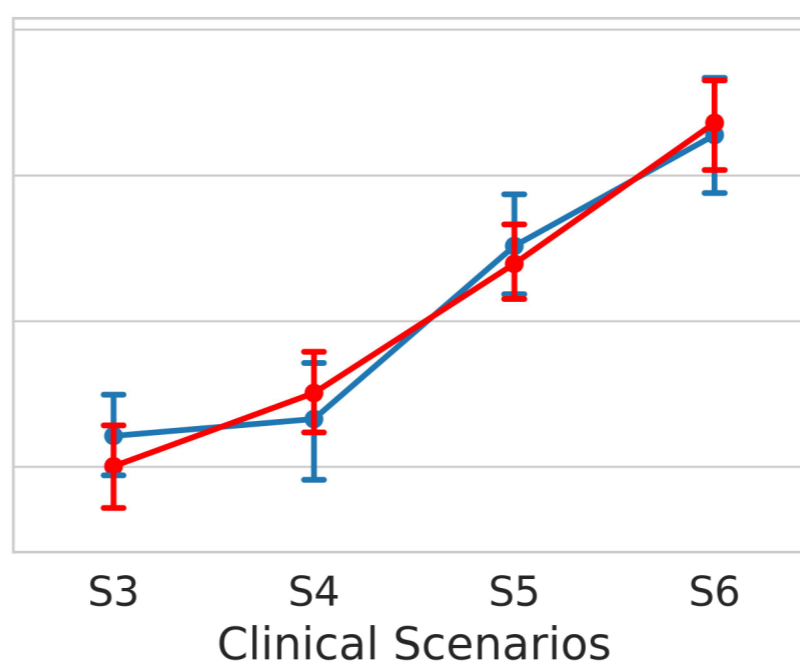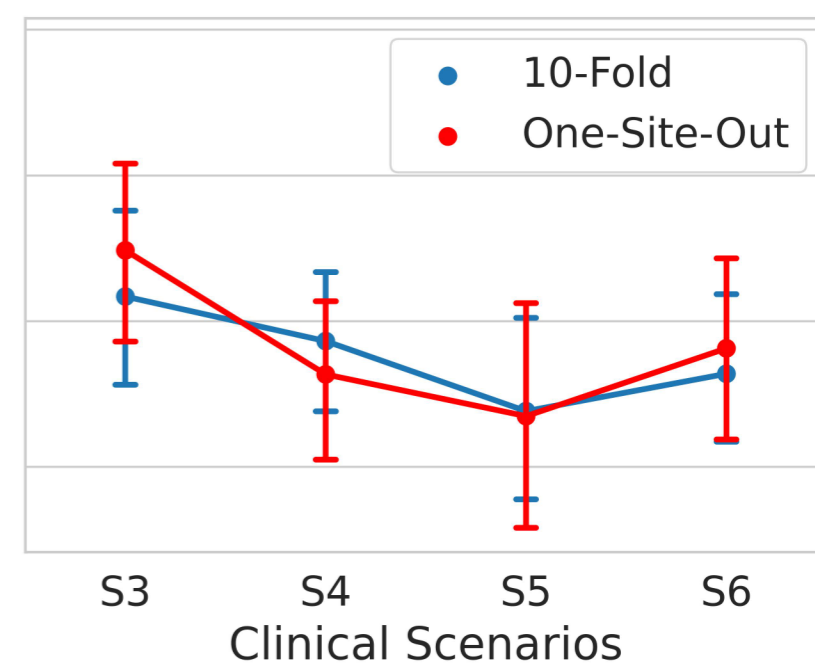

Supplement: Supplementary file 4 — Figure S4. Balanced accuracies (BACs) of the model across three outcome measures (first column: symptomatic remission, second column: clinical global remission, and third column: functional remission) for six clinical scenarios. The x‐axes represent the clinical scenarios in phase one (S1 and S2) and phase 2 of the study (S3, S4, S5, and S6). The y‐axis shows the BAC. The blue and red lines represent the results for 10‐fold and one‐site‐out cross‐validation, respectively. The error bars show the standard deviation of performance across 20 repetitions. The results in the first row show the BAC in phase one in a 4‐week prediction. The added use of time point W1 increases the BAC for all outcome measures. This is mainly due to the increased sensitivity of the model when a new time point is added. The second row shows the results of phase two in a 10‐week prediction. Except for one instance (functional remission), each added time point further increases the BAC for all outcome measures. The increase in the BACs in this case is a byproduct of the increased specificity (see sFigures [Link], [Link]) of the model when a new time point is added. [file ACPS-151-280-s010.pdf]

Symptomatic Remission

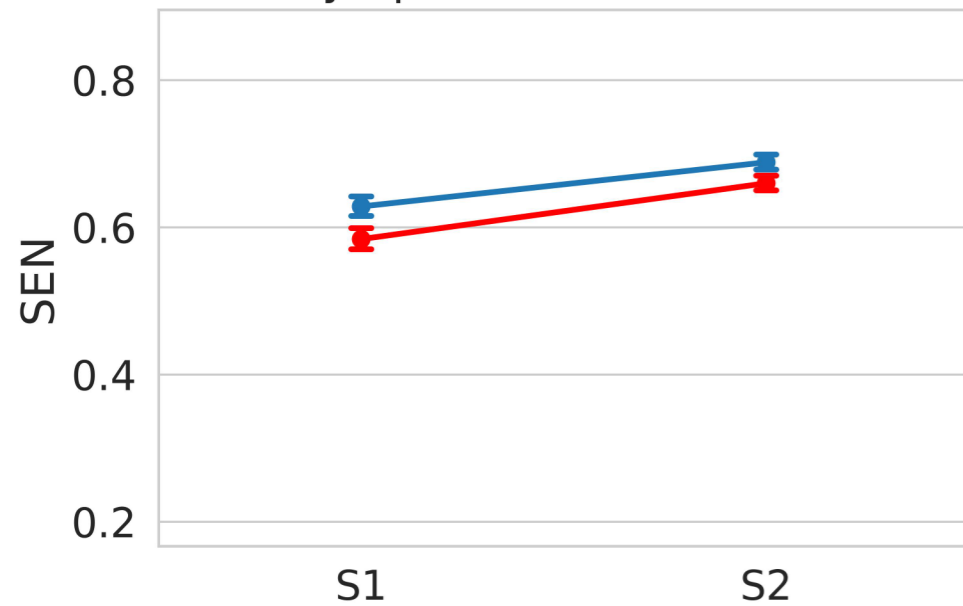

Clinical Global Remission

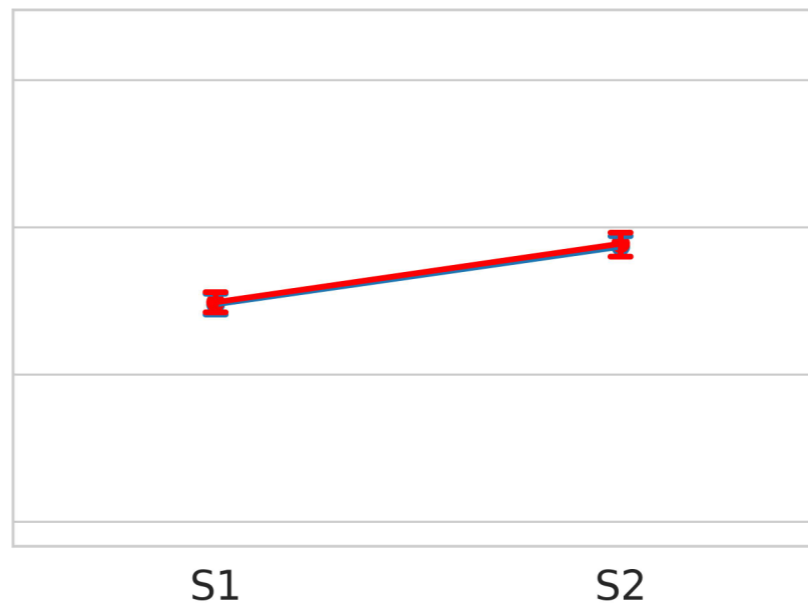

Functional Remission

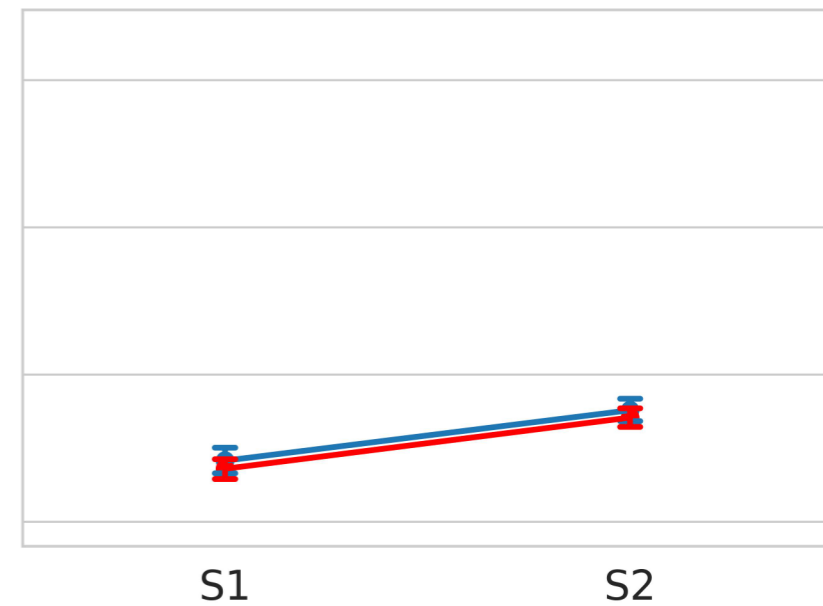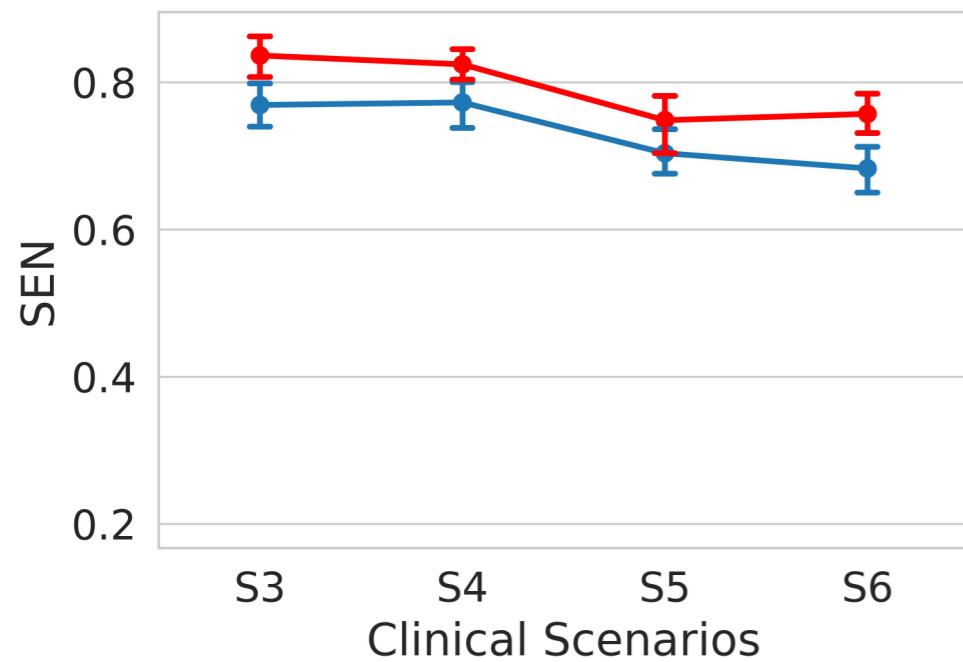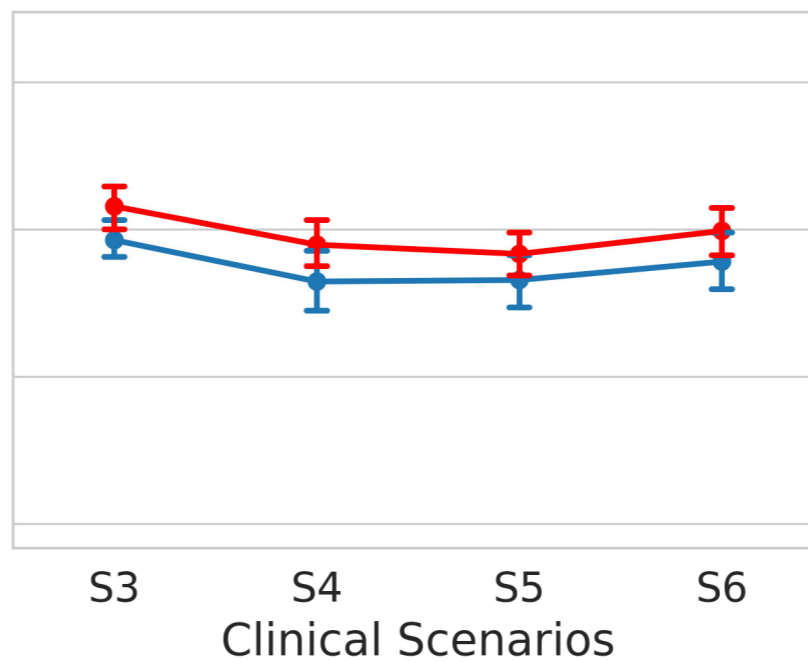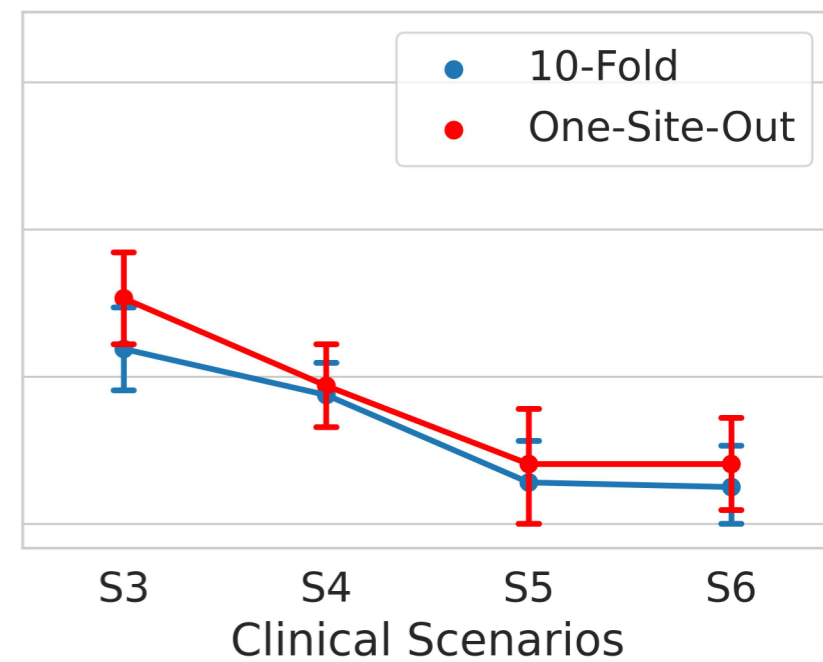

Supplement: Supplementary file 5 — Figure S5. Sensitivity (SEN) of the model across three outcome measures (first column: symptomatic remission, second column: clinical global remission, and third column: functional remission) for six clinical scenarios. The x‐axes represent the clinical scenarios in phase one (S1 and S2) and phase two of the study (S3, S4, S5, and S6). The y‐axis shows the SEN. The blue and red lines represent the results for 10‐fold and one‐site‐out cross‐validation, respectively. The error bars show the standard deviation of SENs across 20 repetitions. The results in the first row show the SEN in phase one in a 4‐week prediction. The added use of time point W1 increases the SEN for all outcome measures. The second row shows the results of phase two in a 10‐week prediction. In most cases adding a new time point results in a reduced sensitivity of the model. [file ACPS-151-280-s004.pdf]

Symptomatic Remission

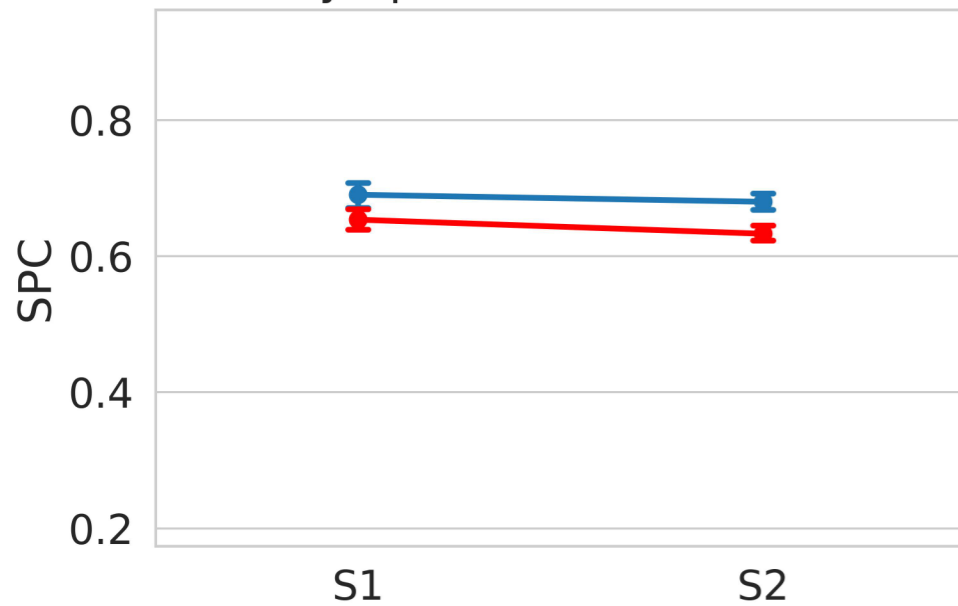

Clinical Global Remission

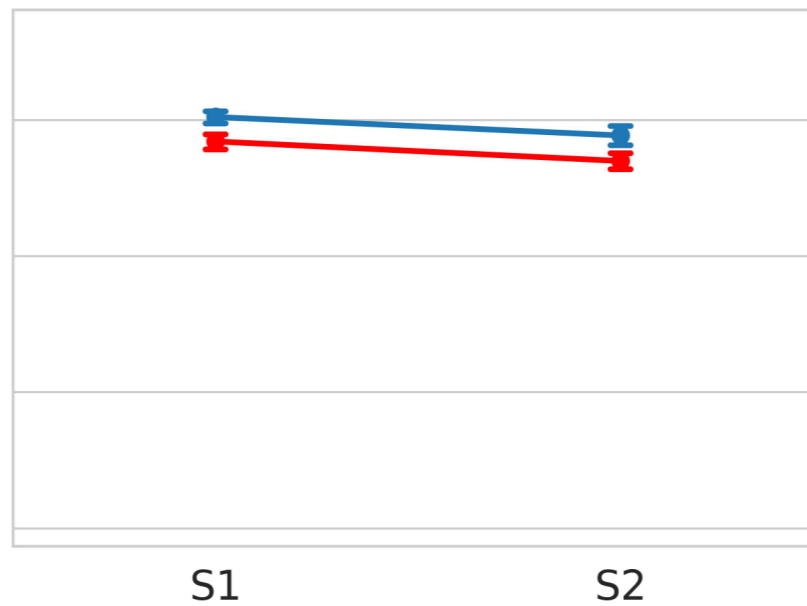

Functional Remission

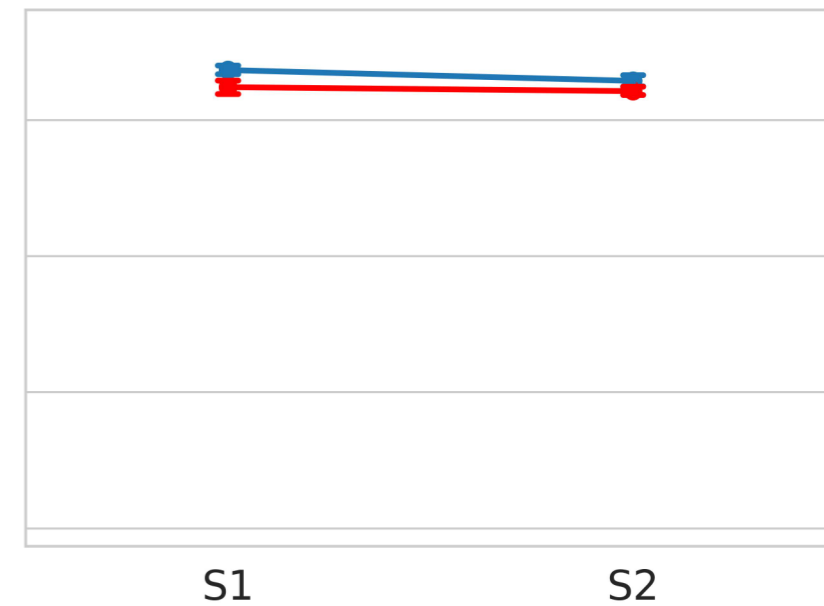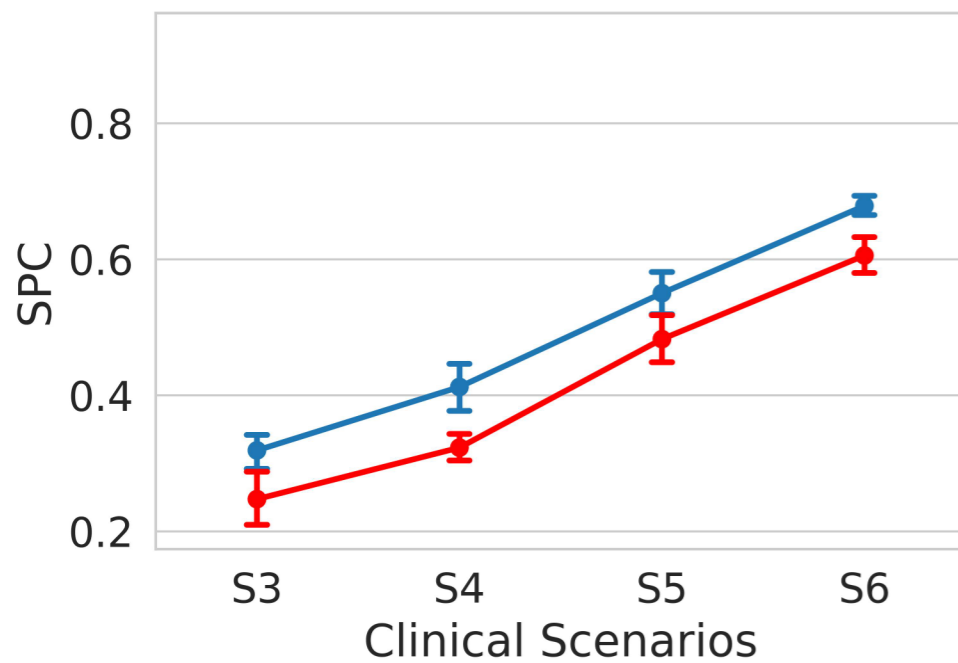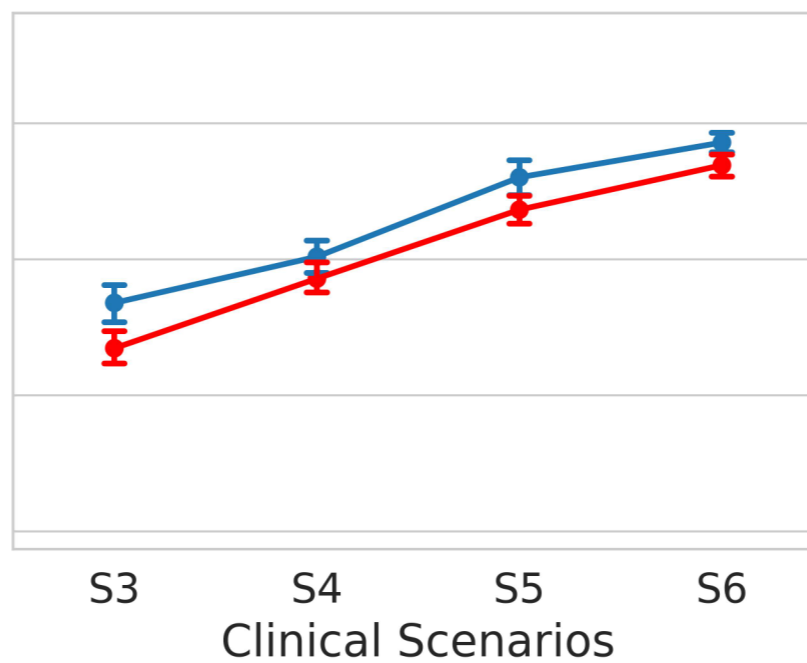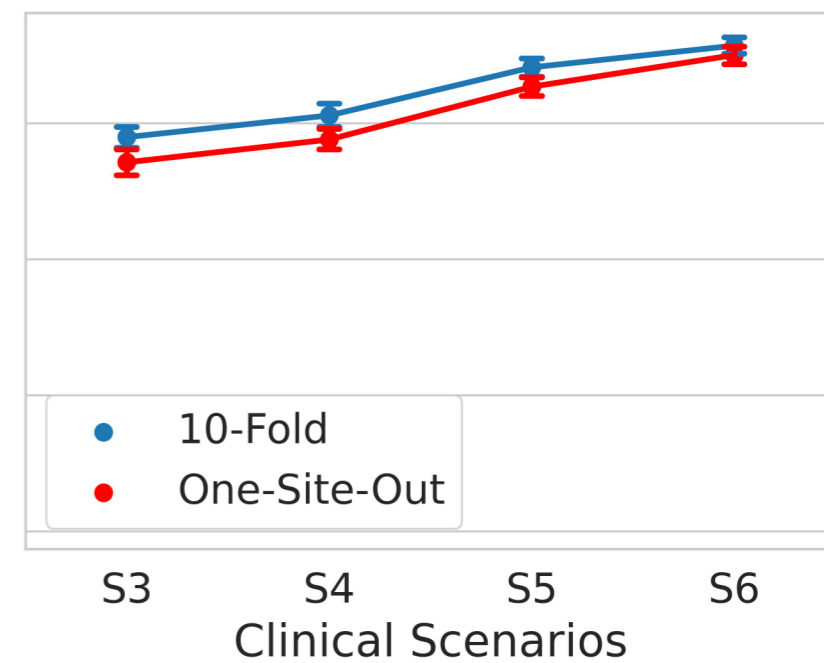

Supplement: Supplementary file 6 — Figure S6. Specificity (SPC) of the model across three outcome measures (first column: symptomatic remission, second column: clinical global remission, and third column: functional remission) for six clinical scenarios. The x‐axes represent the clinical scenarios in phase one (S1 and S2) and phase two of the study (S3, S4, S5, and S6). The y‐axis shows the SPC. The blue and red lines represent the results for 10‐fold and one‐site‐out cross‐validation, respectively. The error bars show the standard deviation of SPCs across 20 repetitions. The results in the first row show the SPC in phase one in a 4‐week prediction. The added use of time point W1 slightly decreases the SPC for all outcome measures. The second row shows the results of phase two in a 10‐week prediction. In all cases adding a new time point results in higher model specificity. [file ACPS-151-280-s012.pdf]

Visit 1

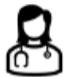

Visit 2

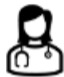

Visit 3

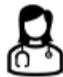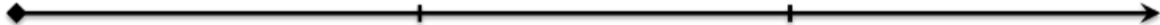

Time

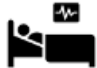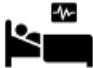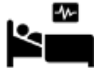

Status 1

Status 2

Status 3

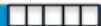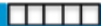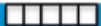

Model 1

Model 2

Model 3

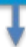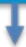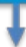

Outcome t

Outcome t

Outcome t

Supplement: Supplementary file 7 — Figure S7. To handle dynamic patient status in outcome prediction using conventional ML approaches, we need specialized models for data collected at each visit. [file ACPS-151-280-s006.pdf]

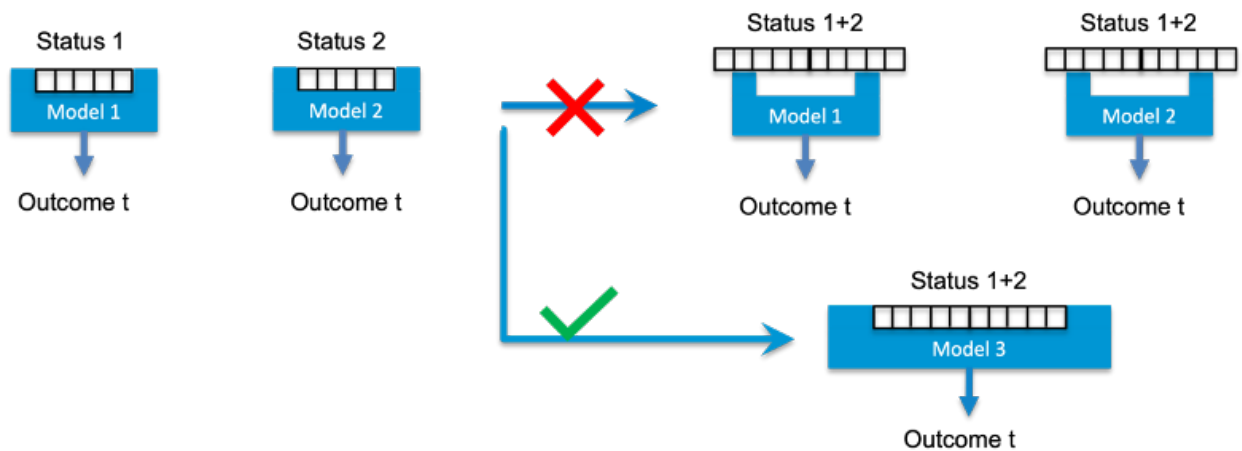

Supplement: Supplementary file 8 — Figure S8. When using conventional ML approaches for outcome prediction, due to their fixed input size, we cannot feed them with accumulated data over time. We need a new model for the mixed data. [file ACPS-151-280-s007.pdf]

Status  $t-1$

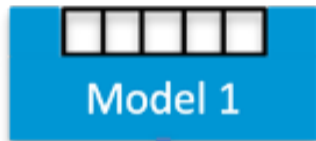

Outcome  $t$

Status  $t-1$

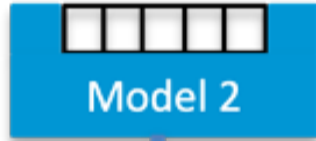

Outcome  $t+1$

Supplement: Supplementary file 9 — Figure S9. Using conventional approaches, we should train several specialized models to accurately predict at different time points in the future. [file ACPS-151-280-s002.pdf]

(a) Single-Task

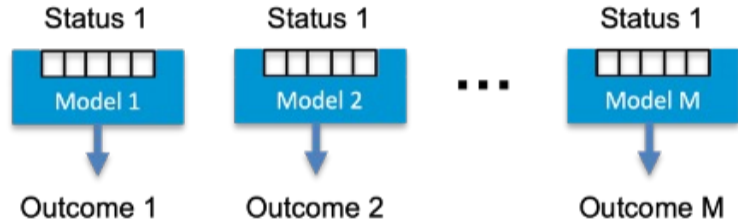

(b) Multi-Task

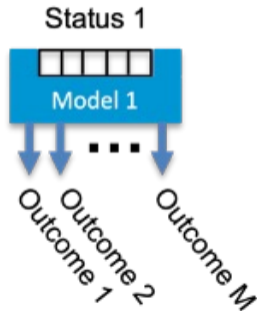

Supplement: Supplementary file 10 — Figure S10. Using conventional single‐task approaches, we need to train one model per outcome. This is while the proposed multi‐task approach can predict several outcomes simultaneously. [file ACPS-151-280-s011.pdf]
