# Supplementary material for: Psychosis Prognosis Predictor: A continuous and uncertainty‐aware prediction of treatment outcome in first‐episode psychosis
Source: Acta Psychiatr Scand. 2024 Sep 18;151(3):280–92. doi: 10.1111/acps.13754 (PMC11787921; doi:10.1111/acps.13754)
Supplement: Supplementary file 11 — Tables S11. Supplementary Tables. [file ACPS-151-280-s005.docx]

**sTable 1.** Balanced accuracy (BAC) of the prediction models predicting three outcome measures (symptomatic remission, clinical global remission, and functional remission) for six clinical scenarios (S_1_-S_6_).

| **Clinical Scenario** | ***N*** | **Symptomatic remission** | | **Clinical global remission** | | **Functional remission** | |
| --- | --- | --- | --- | --- | --- | --- | --- |
| **BAC** | | **10-fold** | **one-site-out** | **10-fold** | **one-site-out** | **10-fold** | **one-site-out** |
| **S_1_** | 371 | 0.659  (0.018) | 0.619  (0.015) | 0.650  (0.015) | 0.633  (0.014) | 0.578  (0.020) | 0.560  (0.012) |
| **S_2_** | 371 | 0.684  (0.014) | 0.646  (0.015) | 0.675  (0.014) | 0.659  (0.018) | 0.604  (0.012) | 0.592  (0.014) |
| **S_3_** | 72 | 0.544  (0.030) | 0.542  (0.027) | 0.561  (0.032) | 0.550  (0.033) | 0.608  (0.069) | 0.624  (0.071) |
| **S_4_** | 72 | 0.592  (0.024) | 0.574  (0.024) | 0.566  (0.049) | 0.575  (0.032) | 0.593  (0.056) | 0.582  (0.066) |
| **S_5_** | 72 | 0.627  (0.032) | 0.615  (0.047) | 0.626  (0.040) | 0.620  (0.031) | 0.569  (0.074) | 0.567  (0.090) |
| **S_6_** | 72 | 0.681  (0.034) | 0.681  (0.030) | 0.664  (0.046) | 0.668  (0.036) | 0.582  (0.058) | 0.591  (0.071) |

The values are averaged over 20 repetitions of 10-fold and one-site-out cross-validation. The values in the parentheses represent the standard deviation over these repetitions.

**sTable 2.** Sensitivity of the prediction models predicting three outcome measures (symptomatic remission, clinical global remission, and functional remission) for six clinical scenarios (S_1_-S_6_).

| **Clinical Scenario** | ***N*** | **Symptomatic remission** | | **Clinical global remission** | | **Functional remission** | |
| --- | --- | --- | --- | --- | --- | --- | --- |
| **SENSITIVITY** | | **10-fold** | **one-site-out** | **10-fold** | **one-site-out** | **10-fold** | **one-site-out** |
| **S_1_** | 371 | 0.628  (0.032) | 0.584  (0.033) | 0.495  (0.032) | 0.498  (0.032) | 0.283  (0.041) | 0.272  (0.031) |
| **S_2_** | 371 | 0.688  (0.023) | 0.660  (0.023) | 0.574  (0.033) | 0.578  (0.038) | 0.352  (0.034) | 0.342  (0.030) |
| **S_3_** | 72 | 0.769  (0.068) | 0.836  (0.065) | 0.585  (0.064) | 0.631  (0.068) | 0.438  (0.138) | 0.506  (0.149) |
| **S_4_** | 72 | 0.772  (0.074) | 0.824  (0.049) | 0.529  (0.092) | 0.579  (0.071) | 0.375  (0.107) | 0.388  (0.134) |
| **S_5_** | 72 | 0.703  (0.071) | 0.748  (0.090) | 0.531  (0.080) | 0.567  (0.068) | 0.256  (0.143) | 0.281  (0.185) |
| **S_6_** | 72 | 0.683  (0.072) | 0.757  (0.064) | 0.556  (0.089) | 0.598  (0.073) | 0.250  (0.128) | 0.281  (0.156) |

The values are averaged over 20 repetitions of 10-fold and one-site-out cross-validation. The values in the parentheses represent the standard deviation over these repetitions.

**sTable 3.** Specificity of the prediction models predicting three outcome measures (symptomatic remission, clinical global remission, and functional remission) for six clinical scenarios (S_1_-S_6_).

| **Clinical Scenario** | ***N*** | **Symptomatic remission** | | **Clinical global remission** | | **Functional remission** | |
| --- | --- | --- | --- | --- | --- | --- | --- |
| **SPECIFICITY** | | **10-fold** | **one-site-out** | **10-fold** | **one-site-out** | **10-fold** | **one-site-out** |
| **S_1_** | 371 | 0.690  (0.044) | 0.653  (0.034) | 0.804  (0.022) | 0.768  (0.026) | 0.873  (0.015) | 0.848  (0.023) |
| **S_2_** | 371 | 0.680  (0.029) | 0.633  (0.026) | 0.777  (0.034) | 0.740  (0.026) | 0.857  (0.018) | 0.842  (0.015) |
| **S_3_** | 72 | 0.319  (0.062) | 0.247  (0.092) | 0.536  (0.063) | 0.469  (0.058) | 0.779  (0.035) | 0.742  (0.044) |
| **S_4_** | 72 | 0.412  (0.084) | 0.323  (0.047) | 0.604  (0.055) | 0.571  (0.051) | 0.811  (0.041) | 0.776  (0.037) |
| **S_5_** | 72 | 0.550  (0.072) | 0.482  (0.085) | 0.720  (0.060) | 0.673  (0.048) | 0.882  (0.032) | 0.853  (0.033) |
| **S_6_** | 72 | 0.678  (0.035) | 0.605  (0.061) | 0.771  (0.035) | 0.738  (0.036) | 0.914  (0.029) | 0.900  (0.032) |

The values are averaged over 20 repetitions of 10-fold and one-site-out cross-validation. The values in the parentheses represent the standard deviation over these repetitions.

**sTable 4.** Comparison between the AUCs of the proposed method with SVM classifiers in six clinical scenarios and when predicting symptomatic remission.

|  | **S_1_** | **S_2_** | **S_3_** | **S_4_** | **S_5_** | **S_6_** |
| --- | --- | --- | --- | --- | --- | --- |
| **SVM** | 0.58 (0.02) | 0.61 (0.01) | 0.56 (0.03) | 0.55 (0.03) | 0.59 (0.03) | 0.74 (0.02) |
| **PPP** | 0.70 (0.01) | 0.73 (0.01) | 0.57 (0.03) | 0.64 (0.03) | 0.67 (0.03) | 0.75 (0.03) |
