# Supplementary material for: Psychosis Prognosis Predictor: A continuous and uncertainty‐aware prediction of treatment outcome in first‐episode psychosis
Source: Acta Psychiatr Scand. 2024 Sep 18;151(3):280–92. doi: 10.1111/acps.13754 (PMC11787921; doi:10.1111/acps.13754)
Supplement: Supplementary file 12 — Text S12. Supporting Information. [file ACPS-151-280-s008.docx]

**Psychosis Prognosis Predictor: A Continuous and Uncertainty-Aware Prediction of Treatment Outcome in First-Episode Psychosis**

Supporting information

**MATERIALS & METHODS**

**The Psychosis Prognosis Predictor**

The proposed architecture, depicted in Figure 1, comprises four conceptual modules that work synergistically to achieve accurate predictions:

1. *Static module:* which is responsible for handling the static input features that remain constant over time. This module preprocesses the static features through a specific procedure tailored to different types of features. For continuous features, the missing data is imputed using the median value of that feature. Subsequently, a robust min-max scaler is applied to rescale the features within the range of [0,1]. To ensure the scaler is robust to outliers, the min and max values are determined based on the median of the bottom and top 10 percent of the feature values, respectively. Binary and categorical features undergo a similar preprocessing procedure. Missing values in binary and categorical features are replaced with the most frequent values observed in the dataset. Categorical features are then subjected to one-hot encoding to represent the different categories, while binary variables are encoded using a simple -1/1 scheme. By employing these preprocessing steps within the static module, the input static features are effectively prepared for integration with the dynamic components of the architecture.
2. *Dynamic module:* which is responsible for processing the input data that changes over time, known as the dynamic features. In this study, the dynamic measures consist of PANSS (30 measures), PSP (5 measures), and CGI (2 measures) assessments. The dynamic module consists of modality-specific long short-term memory (LSTM) units, a recurrent neural network architecture^25^, that is well suited for making predictions on time series data^26,27^. The number of neurons in each LSTM unit is determined by multiplying the number of measures in each assessment by 2; thus, 60 neurons for PANSS, 10 neurons for PSP, and 4 neurons for CGI. The LSTM units serve as the representation learning layer within the dynamic module. They receive the dynamic features starting from the baseline (W0) up to a user-defined endpoint *t*. The LSTM units process the dynamic features and transform them into time-varying middle representations of dynamic data, denoted as Z1, Z2, and Z3 for PANSS, PSP, and CGI measures, respectively. After the dynamic features are transformed into these learned representations, they are merged with the static features in the fusion layer. This fusion step combines static and dynamic information, resulting in a comprehensive set of features. The fused features are then forwarded to the subsequent layers in the regression and classification modules for further analysis and prediction.
3. *Regression module:* which receives the outputs of the static and dynamic modules to predict the dynamic data at the next time point *t*+1. It takes the merged static features and learned dynamic representations as inputs and employs modality-specific two-layer dense layers with ReLU (rectified linear unit) transfer function for this mapping. The first layer in the regression module is called the interaction layer. It consists of time-distributed dense layers that have 30, 10, and 4 neurons respectively for PANSS, PSP, and CGI measures. The purpose of this layer is to capture the potential interactions between different static and dynamic data modalities. The number of neurons in this layer is determined based on the number of features in each modality, allowing for the computation of residuals. To compute the residuals, the outputs of the interaction layers are subtracted from the actual PANSS, PSP, and CGI values in the inputs. The second layer in the regression module is the output layer, which predicts the dynamic features at the next time point. By being able to predict the dynamic features of the subsequent time point, the model gains the capability to predict the future indefinitely. This is achieved by concatenating the predicted outputs with the dynamic inputs and feeding them back into the network for predicting the measures at *t+2* (the thick yellow arrow in Figure 1). This recursive procedure can be utilized to predict the model's output at time *t+k,* where *k* represents an arbitrary time point in the future, given the data from time 0 to time *t*.
4. *Classification module:* this receives the same inputs as the regression module and predicts the probability of target classes (not-remitted or remitted) at time *t+*1 for three outcome measures namely symptomatic remission (SR), clinical global remission (CR), and functional remission (FR). In the interaction layer of the classification module, task specific and time-distributed dense layers with 5 neurons and ReLU transfer functions are utilized to capture the interactions between features. The number of neurons in this layer is predetermined prior to experiments to ensure a fair evaluation and does not undergo further optimization. This layer aims to exploit the potential interactions between different features. The output layer consists of *softmax* layers with two neurons for each task. These neurons predict the probability of the target classes for each outcome measure, distinguishing between the remitted and not-remitted states. By employing a multi-task architecture in the classification module, the network can leverage the covariance structure that may exist among the outcome variables. To enhance the reliability of the predicted probabilities in the classification module, isotonic regression is applied to the training data. This additional calibration process allows for the direct interpretation of the predicted probabilities as a confidence level for each class in the outcomes and improves the reliability of the confidence estimates.

**Pretraining, data augmentation, and training**

The proposed architecture has 39,870 parameters, while the number of available samples in the OPTiMiSE dataset is ~400 patients. This makes the optimization process challenging. To address this problem, we used pretraining and data augmentation techniques. In this direction, we first simulated the data for 10000 patients by randomly drawing from the range between the minimum and maximum values for each feature. Then, we pre-trained the network using simulated synthetic data. For data augmentation, we augmented the samples in the training set using a variable-length sliding window. sFigure 1 illustrates the data augmentation procedure in which 10 samples with time lengths of 2, 3, 4, and 5 are derived from one sample with a time length of 5.

A multi-objective loss function is optimized during the training procedure in which weighted mean squared and categorical cross-entropy loss functions are respectively used for regression and classification tasks. We used 1 and 5 as weights respectively for regression and classification loss functions. An Adam optimizer with an initial learning rate of *3* × *10*^−^*^4^* was employed during the optimization procedure. The exponential learning rate decay with a decay rate of 0.9 and decay steps of 10000 is used. In the pretraining phase, we trained the network for two epochs with a mini-batch size of 25 samples. In the training phase, the network is trained on augmented data for 50 epochs with a mini-batch size of 2 samples.

**From predictions to uncertainty-aware clinical decisions**

Fuzzy logic provides a mathematical framework for representing vague and imprecise information. We employ Mamdani's rule-based fuzzy inference procedure^30^ in four steps:

1. *Uncertainty interval estimation:* 100 repetitions of the Monte-Carlo dropout technique^35^ are used to estimate the uncertainties in predictions of the classification module. The median value across these 100 predictions is used as the estimated probability of remission (*p*). Then, the robust minimum and maximum (median of 10% lowest/highest) predicted probabilities are calculated to estimate the worst-case and the best-case probability of remission (*p_w_* and *p_b_*), respectively. In fact, *p_b_* minus *p_w_* represents the uncertainty interval for a certain prediction.
2. *Fuzzification of inputs and outputs:* Fuzzification is the process of translating numerical values into linguistic variables. This is performed via fuzzy membership functions that assign a degree of membership (between 0 and 1) to each numerical value. In our specific application, we define 5 Gaussian membership functions (sFigure 2a) for the predicted probabilities (*p*, *p_w_*, and *p_b_*), including $G(0,\sigma)$ for ‘very low’, $G(0.25,\sigma/2)$ for ‘low’, $G(0.5,\sigma)$ for ‘medium’, $G(0.75,\sigma/2)$ for ‘high’, and $G(1,\sigma)$ for ‘very high’. Furthermore, 7 Gaussian membership functions (sFigure 2b) are used to categorize the outcome decisions into 7 categories, including $G(0,\sigma/2)$ for ‘definite no-remission (DN)’, $G(0.2,\sigma/2)$ for ‘probable no-remission (PN)’, $G(0.4,\sigma/2)$ for ‘unsure no-remission (UN)’, $G(0.5,\sigma/4)$ for ‘unsure (US)’, $G(0.6,\sigma/2)$ for ‘unsure remission (UR)’, $G(0.8,\sigma/2)$ for ‘probable remission (PR)’, and $G(1,\sigma/2)$ for ‘definite remission (DR)’. In our experiments, since we have 7 decision categories, we heuristically set $\sigma=1/7=0.143$.
3. *Fuzzy inference:* where a set of seven if-then rules are used to translate the membership values for input probability of remissions (*p*, *p_w_*, and *p_b_*) into membership values for seven decision categories (DN, PN, UN, US, UR, PR, and DR). The logical *‘and’* and *‘or’* operations within the rules are respectively implemented using fuzzy *‘max’* and *‘min’* operators. sFigure 3 depicts the fuzzy inference procedure using these seven if-then rules when *p*=0.90, *p_w_*=0.25, and *p_b_*=1.00. For example, the first rule says ‘if the probability of remission is very high and the worst-case probability of remission is high or very high, then the decision is definite remission’. The membership value of a very high probability of remission for *p*=0.90 is 0.78, and membership values of the high and very high worst-case probability of remission for *p_w_*=0.25 are approximately 0. Thus, the membership value for the definite remission decision is $min(0.78, max(0,0))=0$. In this example, PR and UR decisions have the highest membership values with 0.21 and 0.78, respectively, and the memberships for the rest categories remain very close to zero. We use the maximum membership value across seven outcome decisions to specify the final decision. In this case, the final decision is unsure remission (UR) which is a reasonable decision given that the predicted probability of remission is high but the model is very uncertain about it.
4. *Aggregation and defuzzification:* to calculate the uncertainty-aware probability of remission ($\tilde{p}$) given the model uncertainty, we first aggregate the membership functions for seven clinical decisions using the fuzzy max aggregation that performs similarly to a union operation. Then in the defuzzification step, we compute the centroid of the aggregated mass and use its *x* coordinate as the modified probability of remission. In our example, the uncertainty-aware probability of remission is 0.65 which shows a significant discount compared to the initial probability of remission of 0.90 given its high uncertainty.

**Model training procedure and evaluation**

The metrics used in this study include:

1. *Area Under the Receiver Operating Characteristic Curve (AUC):* This metric quantifies the overall discriminative power of the model. It represents the ability of the model to distinguish between the positive and negative classes.
2. *Balanced Accuracy (BAC):* BAC takes into account both sensitivity and specificity and provides a balanced measure of the model's accuracy. It is particularly useful when the dataset is imbalanced, meaning the number of samples in each class is significantly different.
3. *Sensitivity:* Sensitivity measures the proportion of true positive predictions out of all actual positive samples. It indicates the model's ability to correctly identify positive cases.
4. *Specificity:* Specificity measures the proportion of true negative predictions out of all actual negative samples. It indicates the model's ability to correctly identify negative cases.

**DISCUSSION**

**Comparison between our approach and more common machine learning models.**

A comprehensive direct comparison is impractical due to inherent limitations in conventional models when addressing the complexity of our study’s scenario. Our manuscript introduces a novel multi-task neural network architecture designed for time-series data to enable uncertainty-aware prediction of multiple outcomes into an unlimited future. This model, once trained, can utilize historical data to forecast various outcomes indefinitely. However, conventional machine learning models face several constraints in this context:

1) **Dynamic Input Size**: Traditional models require a fixed input size and cannot adapt to dynamic changes in patient states over time. For example, a model built to predict an outcome based on the patient’s status at the first medical intervention cannot be applied directly to a later status without modification. For example, if the patient status is defined by 10 variables at time point 1, an SVM model trained to predict outcome at time point t given data at time point 1 can accept 10 variables as its inputs. When having access to the data at time point 2 (another visit), it is not possible to feed the merged input variables (20) to the model. Instead, we need to train another model either i) specialized to predict outcomes using the patient status at time point 2 (see sFigure 7), or ii) with 20 inputs (see sFigure 8).

2) **Single Time-point Prediction**: Conventional models are typically trained to predict outcomes at a specific future time point. Extending these predictions over continuous future time points would require multiple models each trained for a specific duration ahead, such as one week, one month, or a year (see sFigure 9).

3) **Single-task Nature**: Most standard classification methods predict only one outcome at a time, unlike our proposed multi-task model which can simultaneously predict multiple outcomes (see sFigure 10).

4) **Uncertainty Estimation**: Estimating prediction uncertainty is cumbersome with traditional models. Our architecture employs Monte Carlo dropout to quantify uncertainty directly, a significant enhancement over the typical approach requiring bootstrapping and training multiple model instances.

The necessity to train and evaluate potentially thousands of models to conduct a fair comparison renders this impractical. More concretely, when using conventional approaches, for predicting treatment outcomes in the time span of weeks in the future and on historical data collected in the past week, we need to train models for ~100 bootstrap repetitions on training data. This translates to models in our experiments for 3 outcome measures predicted for the next 3 time points (W_4_, W_6_, and W_10_) using past historical data in the past 4 time points (W_0_, W_1_, W_4_, and W_6_). This is where the novelty of our method lies that makes the prediction of multiple outcomes in diverse scenarios possible only using one model.

Even though due to the mentioned reasons a direct head-to-head comparison is not possible, we conducted another experiment with six SVM classifiers corresponding to six clinical scenarios and in predicting symptomatic remission. A repeated (10 times) 10-fold cross-validation is used for evaluation. The results are summarized in sTable 4. The results show the superiority of the proposed PPP model in all six scenarios.

**Limitations**

Reducing the complexity of input features is a valid consideration for improving model efficiency and interpretability. However, several challenges arise with the implementation of feature selection in the context of our study:

- Variability in Feature Relevance: Our dataset's limited size coupled with the necessity of using nested cross-validation introduces significant variability in feature selection across different folds of the external loop. We observe that even minor perturbations in the data can lead to substantial changes in the identification of 'optimal' features. This variability suggests that the stability of selected features is not guaranteed across different subsets of data, which could undermine the reliability of the feature selection process.
- Concerns about Realism and Stability: Due to the noted variability, there is a concern that relying on feature selection might not necessarily result in a more realistic algorithm for clinical practice. The potential for selected features to vary significantly under different clinical and demographic settings could limit the generalizability and robustness of the model.

To address these concerns and enhance the clinical utility of our algorithm without compromising its adaptability and robustness, we are exploring alternative approaches. Currently, we are investigating innovative solutions that enable neural networks to accommodate data with missing values without the need for imputation. One promising avenue is detailed in our recent research (see https://arxiv.org/abs/2206.01640), which outlines a novel method for integrating incomplete data directly into the predictive modeling process.

**FIGURE LEGENDS**

**sFigure 1.** The data augmentation process. A set of ten samples with time-length 2 to 5 are generated for a sample with the length of five timepoints.

**sFigure 2.** a) Five Gaussian membership functions for the probability of remission. These functions are used to map the values of the probability of remission (*p*), the worst-case probability of remission (*p_w_*), and the best-case probability of remission (*p_b_*) in the x-axis to a membership value (between 0 and 1) in the y-axis for ‘very low’, ‘low’, ‘medium’, ‘high’, and ‘very high’ categories; b) Gaussian membership functions for seven clinical decisions, ‘definite no-remission (DN)’, probable no-remission (PN)’, ‘unsure no-remission (UN)’, ‘unsure (US)’, ‘unsure remission (UR)’, ‘probable remission (PR)’, ‘definite remission (DR)’.

**sFigure 3.** Seven rules in the proposed fuzzy inference system for translating the predicted probability of remission (*p*), the worst-case probability of remission (*p_w_*), and the best-case probability of remission (*p_b_*) into risk-aware clinical decisions. The green stars show the value of the corresponding membership function in each rule for an example prediction with *p*=0.9, *p_w_*=0.25, and *p_b_*=1.00. The orange and blue boxes represent the fuzzy max and min operations, respectively. The gray area in the last right column shows the mass under the membership function of each decision. These masses are combined using fuzzy max aggregation. The x-coordinate of the centroid of the aggregated mass represents the uncertainty-aware probability of remission ($\tilde{p}$) that aggregates the model uncertainty into the final prediction.

**sFigure 4. Balanced accuracies (BACs) of the model across three outcome measures (first column: symptomatic remission, second column: clinical global remission, and third column: functional remission) for six clinical scenarios.** The x-axes represent the clinical scenarios in phase one (S_1_ and S_2_) and phase 2 of the study (S_3_, S_4_, S_5_, and S_6_). The y-axis shows the BAC. The blue and red lines represent the results for 10-fold and one-site-out cross-validation, respectively. The error bars show the standard deviation of performance across 20 repetitions. The results in the first row show the BAC in phase one in a 4-week prediction. The added use of time point W_1_ increases the BAC for all outcome measures. This is mainly due to the increased sensitivity of the model when a new time point is added. The second row shows the results of phase two in a 10-week prediction. Except for one instance (functional remission), each added time point further increases the BAC for all outcome measures. The increase in the BACs in this case is a byproduct of the increased specificity (see sFigure 5 and sFigure 6) of the model when a new time point is added.

**sFigure 5. Sensitivity (SEN) of the model across three outcome measures (first column: symptomatic remission, second column: clinical global remission, and third column: functional remission) for six clinical scenarios.** The x-axes represent the clinical scenarios in phase one (S_1_ and S_2_) and phase two of the study (S_3_, S_4_, S_5_, and S_6_). The y-axis shows the SEN. The blue and red lines represent the results for 10-fold and one-site-out cross-validation, respectively. The error bars show the standard deviation of SENs across 20 repetitions. The results in the first row show the SEN in phase one in a 4-week prediction. The added use of time point W_1_ increases the SEN for all outcome measures. The second row shows the results of phase two in a 10-week prediction. In most cases adding a new time point results in a reduced sensitivity of the model.

**sFigure 6. Specificity (SPC) of the model across three outcome measures (first column: symptomatic remission, second column: clinical global remission, and third column: functional remission) for six clinical scenarios.** The x-axes represent the clinical scenarios in phase one (S_1_ and S_2_) and phase two of the study (S_3_, S_4_, S_5_, and S_6_). The y-axis shows the SPC. The blue and red lines represent the results for 10-fold and one-site-out cross-validation, respectively. The error bars show the standard deviation of SPCs across 20 repetitions. The results in the first row show the SPC in phase one in a 4-week prediction. The added use of time point W_1_ slightly decreases the SPC for all outcome measures. The second row shows the results of phase two in a 10-week prediction. In all cases adding a new time point results in higher model specificity.

**sFigure7.** To handle dynamic patient status in outcome prediction using conventional ML approaches, we need specialized models for data collected at each visit.

**sFigure8.** When using conventional ML approaches for outcome prediction, due to their fixed input size, we cannot feed them with accumulated data over time. We need a new model for the mixed data.

**sFigure9.** Using conventional approaches, we should train several specialized models to accurately predict at different time points in the future.

**sFigure10.** Using conventional single-task approaches, we need to train one model per outcome. This is while the proposed multi-task approach can predict several outcomes simultaneously.
